# Assessing the clinical utility of inertial sensors for home monitoring in Parkinson’s disease: a comprehensive review

**DOI:** 10.1038/s41531-024-00755-6

**Published:** 2024-08-20

**Authors:** Stefano Sapienza, Olena Tsurkalenko, Marijus Giraitis, Alan Castro Mejia, Gelani Zelimkhanov, Isabel Schwaninger, Jochen Klucken

**Affiliations:** 1 Luxembourg Centre for Systems Biomedicine (LCSB), University of Luxembourg, Esch-sur-Alzette, Luxembourg; 2 Luxembourg Institute of Health (LIH), Strassen, Luxembourg; 3 Centre Hospitalier de Luxembourg (CHL), Rollengergronn-belair-nord, Luxembourg

**Keywords:** Parkinson's disease, Translational research

## Abstract

This review screened 296 articles on wearable sensors for home monitoring of people with Parkinson’s Disease within the PubMed Database, from January 2017 to May 2023. A three-level maturity framework was applied for classifying the aims of 59 studies included: demonstrating technical efficacy, diagnostic sensitivity, or clinical utility. As secondary analysis, user experience (usability and patient adherence) was evaluated. The evidences provided by the studies were categorized and stratified according to the level of maturity. Our results indicate that approximately 75% of articles investigated diagnostic sensitivity, i.e. correlation of sensor-data with clinical parameters. Evidence of clinical utility, defined as improvement on health outcomes or clinical decisions after the use of the wearables, was found only in nine papers. A third of the articles included reported evidence of user experience. Future research should focus more on clinical utility, to facilitate the translation of research results within the management of Parkinson’s Disease.

## Introduction

Over the last 20 years, the digital transformation of medicine has evolved tremendously due to the development of wearable sensors as clinical support tools providing objective (digital) outcomes for clinical research and in remote monitoring applications for clinical care. The unwavering research interest in wearable sensors relies on their ability to generate accurate, quantitative, real-world, rater-independent, and user-derived measures for various medical applications.

Wearable sensors, or simply “wearables”, are not limited to a specific clinical setting, allowing for continuous or high sampling frequency assessments that are unsustainable by standard clinical evaluations. Consequently, the data generated by wearables can track the whole spectrum of changes in the user’s functional state and link impairment to clinical symptoms of patients. Furthermore, wearables can capture subtle, prodromal, or granular variations in motor symptoms that are clinically relevant but extremely difficult, if not impossible, to detect with current evaluation methods^1,2^. Finally, wearable technologies can be synergized with medical profiling, data mining, and machine learning to generate individualized health reports that inform patients, healthcare professionals, and society. Extracted data can complement patient-reported outcomes with digital objective biomarkers promptly detecting symptom deterioration. The recorded diagnostic information can support healthcare professionals in decision-making and provide a closed feedback loop during interventions. Finally, they can be used as objective and evidence-based indicators to regulate reimbursement within the healthcare ecosystem, providing additional value for payers and policymakers.

Parkinson’s Disease (PD) is an ideal target for the application of wearable sensors. It is the second-most common progressive neurodegenerative disease of the central nervous system^3,4^, known for its diverse clinical phenotypes, affecting both motor and non-motor domains. The former is mainly characterized by bradykinesia, rigidity, tremor, gait and balance impairment, while nonmotor symptoms expand across autonomic, neuropsychiatric, sensory and sleep domains, which may be attributed to aging rather than the underlying pathological mechanism^5^. For patients living with PD, this often means a confrontation with a variety of symptoms in their daily activities, a change in quality of life and interpersonal relationships, and in later life, the need for continuous care^6^. The progressive nature of PD and its heterogeneity across different patients makes the management complex and multi-dimensional^5^. For this reason, the medical workups are also constantly evolving in parallel with the patient’s journey, trying to balance short-term benefits with the long-term effects on the progression of the disease.

In this context, accurately tracking PD symptoms and their oscillation is crucial for optimal care. Typically, this is done during clinical assessments, where the patient is asked to perform a series of standardized tasks. At the same time, a trained neurologist visually evaluates the movements and provides a score for each symptom. In parallel, the non-motor sphere is evaluated via different questionnaires to obtain a comprehensive picture of the subject’s conditions.

However, recent studies have demonstrated that motor symptom severity can fluctuate rapidly, even multiple times in an average 30-min consultation^2^. Consequently, due to the excessive granularity needed, accurate tracking in time is not feasible through standard-of-care methods. These traditional methodologies, such as motor diaries and standard clinical examinations, need to be revised to decrease the rater-dependent variance and subjectivity, low accuracy, low sampling frequency, and low sensitivity-to-change^7,8^. For this reason, wearables raise the expectation that they will complement classical clinical examination, as they provide healthcare professionals with objective measures that may support clinical decisions or yield insights into the efficacy of clinical interventions.

PD is an ideal target for the application of wearables due to the variety of symptoms that appear throughout the stages of the disease (diagnostic target symptom of wearables) and their sensitivity to change following an intervention (monitoring target symptom for wearables)^9^. Monitoring target symptoms through wearables may help detect symptoms fluctuating over different timeframes ranging from months to years (progression of the disease), days to months (treatment kinetics), or even within minutes (motor fluctuations, fast-responding interventions). Thus, in remote monitoring settings, wearables must accurately measure, linking the information recorded with relevant clinical aspects and ultimately providing support to patients or healthcare professionals^10^.

During the last decades, different types of wearable sensors have been developed by researchers to address the aforementioned challenges. Inertial Measurement Units (IMUs) have found an immediate application in investigating motor symptoms such as tremor, dyskinesia, bradykinesia, and mobility impairments. Pressure sensors have also been used to evaluate gait and balance disturbances of patients. Furthermore, wearables have been utilized to better understand the impact of PD on biopotential signals, generating electrocardiograms, electromyograms, and electroencephalograms. Finally, in recent years, electrochemical biosensors have been gaining popularity to monitor α-synuclein and other biomarkers of the disease from blood samples^11^.

Wearables can accurately collect a huge amount of relevant medical information from patients. However, from a practical point of view, their application in the management of PD is still limited.

**Fig. 1:**
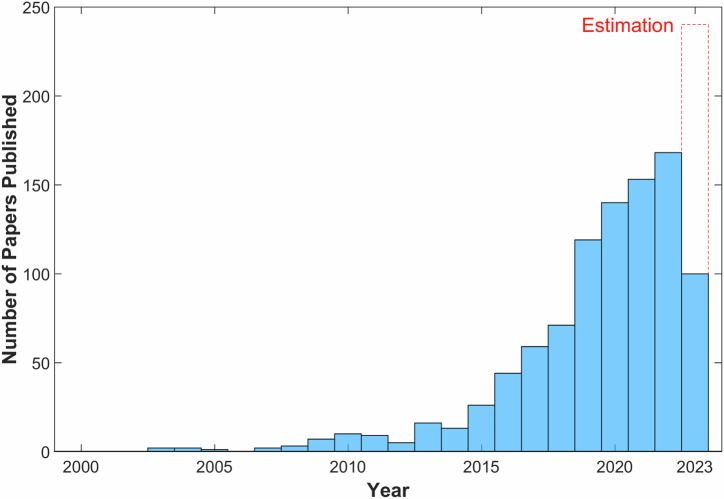
Publication trend of studies focusing on PD and wearables. Number of articles published from 2000 to May 2023 focusing on wearables sensors and PD (screening search terms: Parkinson and wearables); screening performed using PubMed as a data-source (RRID: SCR_004846). In dashed red line the estimation for the full 2023. In total the research revealed 950 papers. This number is expected to exceed one thousand unit by the end of the year.

Looking at the literature on wearables and PD as a joint research topic, since 2000, exclusively in the PubMed Central database (RRID: SCR_004846), 950 papers related to wearables and PD have been published (see Fig. 1). This number is expected to exceed one thousand units by the end of 2023. However, when looking at healthcare procedures, a barrier to the broader implementation of wearables in PD management becomes evident. In real-world care, treatment adjustments are generally based exclusively on doctors’ experience, relying on information collected during short in-person neurological visits. Even though some wearables reached the maximum “Technological readiness” level TRL9 and have gained regulatory approval^12^, their precise scope within PD management remains unclear^13^.

One of the possible causes of this translational gap is a lack of what in this manuscript is called “clinical utility evidence”^14^ defined as: “*an improvement on health outcomes, diagnosis, treatment management or prevention demonstrated after the use of the wearable sensor*”. In fact, the value of wearables for patients and doctors is ultimately determined by the improvements they can generate in terms of health outcomes and treatment management, rather than the technical accuracy of their measurements only. Interestingly, different evidence evaluation frameworks have already conceptualized this *“clinical utility”* concept while addressing the development phases of diagnostic tests in healthcare^15^ or aspects of digital transformation of technology in general^16^.

In general, clinical utility should be measured within the standard of care. However, quantifying the impact of wearables in this context may be extremely challenging, due to the huge variability in terms of patients, settings, care pathways, and healthcare professionals. Research studies can provide a valuable option to reduce this heterogeneity. In fact, they present more structured assessment protocols and inclusion/exclusion criteria, which allow to investigate more easily the clinical utility of these technologies.

For this reason, in this review, we want to evaluate the clinical utility evidences generated by research studies that focus on wearable sensors in PD applied to the home monitoring setting. The final goal is not only clarifying their applicability in term of disease management strategy, but also to better quantify the impact of these technologies on clinical decisions and their effectiveness on improving health outcomes. For this reason, we tailored the existing evidence models towards the evaluation of clinical utility of wearables and reviewed the included studies based on their maturity level. We narrowed our search to articles targeting wearables for home monitoring in people with PD.

In our review process, we categorized eligible articles on wearables by three hierarchical efficacy evidence levels: technical efficacy, diagnostic sensitivity, and clinical utility.

In addition, as secondary analysis, we also evaluated user-experience related aspects presented in the studies. This because the health outcomes obtained might be deeply influenced by the usability of the system and the concordance/adherence of the patients. A detailed description of the methods that we applied, including our evaluation framework and article assignment criteria, can be found in the final section of this review.

## Results

### Article selection

Within the large spectrum of studies on wearable sensors in PD, we identified 296 articles addressing their home monitoring applicability. From these articles, we identified through PubMed between January 2017 and May 2023, 59 studies were eligible for inclusion after screening and full-text eligibility assessment. Interestingly, albeit no exclusion criteria were applied on the type of sensor technology, only a limited number of papers adopted alternative measurement tools to inertial measurement units. More precisely, surface electromyography, pressure sensors and audio stimuli, were utilized in one study each, while 2 articles used GPS to track participants mobility.

**Fig. 2:**
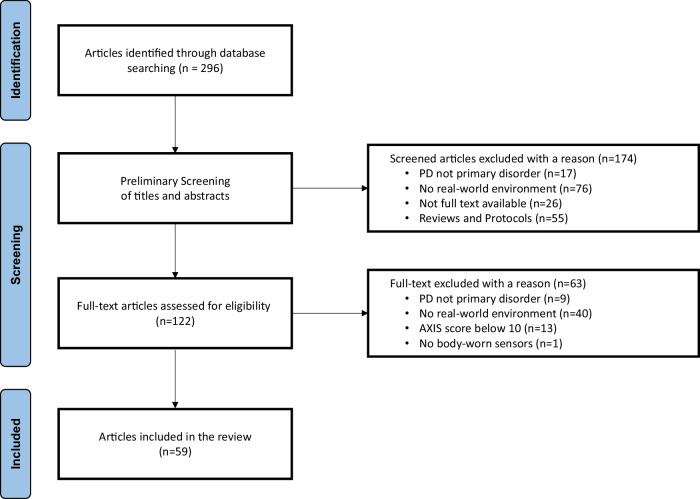
PRISMA flow diagram. Articles screening procedure, including the number of papers excluded during preliminary and full text screening.

The PRISMA flow diagram generated through the review process can be found in Fig. 2, with an overview of excluded studies^17^.

### Article assignment

**Fig. 3:**
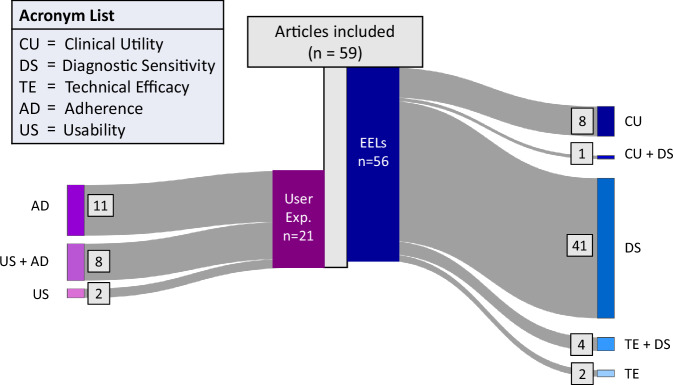
Distribution of articles reviewed. Sankey diagram of the distribution of the articles across the different Efficacy Evidence Levels and User Experience categories.

Articles were assigned to the EELs based on their research questions. The distribution of the papers reviewed, represented in Fig. 3, shows that 56 studies out of 59 included evidence results, while three papers focused exclusively on wearables usability and/or adherence^18–20^. Five articles were assigned in multiple EELs due to overlapping aspects being evaluated: four were assigned to both *Technical Efficacy* and *Diagnostic Sensitivity* levels^21–24^, and one was assigned to *Diagnostic Sensitivity* and *Clinical Utility*^25^. Regarding *User Experience*, 21 articles reported evidence of usability and adherence within their results, and two exclusively assessed the usability level^26,27^. The using environment of the research studies analyzed in this review is the home setting. However, in the supplementary tables of this manuscript we also reported separately the results obtained in lab environment, when present.

### Level I. technical efficacy

Research questions on *technical efficacy* were investigated in six articles^21–24,28,29^. They assessed the accuracy of sensor-based mobility measures without connecting the outcomes to clinical scores or patient phenotypes. Overall, the primary focus of EEL-I articles was to detect movements of interest in a real-world environment, such as walking bouts and activities of daily living. The most common goal shared by three out of the six articles was accurately detecting gait segments in the unsupervised environment^23,28,29^.

Patient numbers varied significantly across studies, with a minimum of four patients^22^ up to a maximum of 25 people with PD^21,23^. Full results are presented in Supplementary Table 1.

### Level II. diagnostic sensitivity

The second level was associated with research questions on *diagnostic sensitivity*. A total of 46 articles were included, which accounted for >75% of those reviewed. Study types and research questions varied significantly across the group, spanning from assessing the capability of gait sensors to differentiate the mobility of PD versus healthy controls or other neurodegenerative diseases^26,30–34^ to comparing sensor-derived parameters with symptoms^23,35–49^ and clinical scores^21,50,51^. Differences across PD patient phenotypes were also investigated across fallers vs. non-fallers^43,44^ and freezers vs. non-freezers^45,46^.

Additionally, three manuscripts presented results using machine learning models for prediction. Shah et al.^31^ and L. Evers et al.^30^, while discriminating PD versus HC, achieved an AUC of 0.89 and 0.76, respectively, while Mancini et al.^45^ classified freezers vs. non-freezers with an AUC equal to 0.90. The impact of the “Real-World” noise was also assessed but difficult to quantify. Powers et al.^25^ reported 8% of false positives in their study due to manual teeth brushing and 2% linked to playing musical instruments. Furthermore, it was observed that measures in the clinic are often not representative of the patient’s conditions at home^41^, as patients’ gait at home was slower, strides shorter, and shuffling gait more present. The remaining results are presented in Supplementary Table 2.

### Level III. clinical utility

Clinical utility as the EEL-III included nine articles (15%)^25,52–59^. No increasing trend was /observed in the six years considered (0 articles until May 2023, 1 paper in 2022, 4 studies from 2021, 2 from 2019, 0 from 2018, and 2 from 2017. See Table S3 for details). Three articles presented interventional studies where remote monitoring wearable sensors improved outcomes through individualized measures within training or rehabilitation settings^57–59^. Two assessed auditory stimuli for mobility training^57,58^. The remaining studies explored more traditional treatment programs^25,52–56^.

The utility of wearables information in the context of clinical decisions was assessed in 6 articles^25,52–56^. The contribution of sensor-based measurements in providing sufficient additional knowledge to justify therapy changes varied across the studies, from 6% (6 out of 100 participants) in^25^ up to 43% (85 out of 200 participants) in ref. ^56^. The medical impact of wearables was quantified in two studies^54,56^, which both observed statistically significant improvements in terms of clinical scores when sensor measurements support doctors and healthcare professionals. Complete study outcomes are listed in Supplementary Table 3.

### Types of efficacy evidence provided

Four macro types of analytical evidence have been used to prove the different EELs. Statistical methods were the most exploited tools to compare groups, diagnoses, and interventions. Primarily, descriptive statistic of the distributions examined was presented together with an associated *p*-value. Rarely were Cohen-d values for the effect size or Area Under the Curve (AUC) values presented.

Correlations analysis, described generally as correlation coefficients and relative *p*-values, were the most common approaches for the analysis of symptom severity. In some cases, confidence intervals (CI) and *R*^*2*^ values were also reported. Intraclass Correlation Coefficient (ICC) was unanimously applied to evaluate test-retest reliability.

Articles based on machine learning metrics, all provided with measures beyond pure accuracy. If the AUC was overall the most frequent outcome, sensitivity, specificity, F1 score, and mean absolute error were used in the different papers reviewed.

Finally, qualitative/descriptive statistics were utilized in the reviewed papers. It is interesting to mention that this type of approach was used only in EEL-I when feasibility was tested or when describing clinical decision support in EEL-III.

For detailed results on the analytical evidence types, see Tables 1, 2, and 3.

**Table 1:** Types of Evidence provided in the EEL-I

Authors study	Reference	Aim	Target	Technical efficacy evidence provided
A. Abrami. et al.	^21^	Feasibility Testing	Testing the feasibility of decomposing movement in the home environment in cluster.	Convergence to transition matrix, variance of symbolic movement representation first PCA.
Ravichandran. et al.	^22^	Feasibility Testing	Test the feasibility to collect fine finger dexterity tasks through an instrument glove with IMUs.	System level qualitative performance.
Y. Raykov. et al.	^23^	Algorithm Validation	Detect gait and non-gait segments from IMU sensors using a machine learning algorithm.	Specificity and sensitivity performance of machine learning algorithm compared to video recordings.
M. Ullrich. et al.	^28^	Algorithm Validation	Automated detection of unsupervised gait task at home using a machine learning algorithm.	F1 score performance of machine learning algorithm compared to manually annotated data.
Y. Brand. et al.	^29^	Algorithm Validation	Detect gait and non-gait segments from IMU sensors positioned on the wrist using a machine learning algorithm.	AUC performance of machine learning algorithm compared to ground truth obtained from a sensor positioned on the back of the participant.
A. Nouriani. et al.	^24^	Algorithm Validation	Detect multiple walking and postural changes from IMU sensors using a machine learning algorithm.	Specificity, sensitivity positive predictive value, negative predictive value, and accuracy performance of machine learning algorithm compared to video recordings.

**Table 2:** Types of evidence provided in the EEL-II

Authors study	Reference	Aim	Target of the study	Diagnostic sensitivity evidence provided
R. Bhidayasiri. et al.	^84^	Treatment monitoring	To evaluate the efficacy of rotigotine transdermal patch in RCT, using a wearable sensor features as final endpoint	Significant differences, determined by statistical test *p*-values, in sensor-derived features before/after intervention
M. Iijima. et al.	^85^	Treatment monitoring	Objectively assessing gait disorder before/after medication change	Significant differences, determined by statistical test *p*-values, in sensor-derived features before/after the treatment change
H. Khodakarami et al.	^86^	Treatment monitoring	Predicting levodopa response using a machine learning algorithm trained on sensor data	AUC performance of machine learning algorithm when predicting 6 different classes of levodopa responses.
R. Bouça-Machado et al.	^87^	Treatment monitoring	To evaluate the efficacy of intervention using a wearable sensor features as final endpoint	Significant differences, determined by statistical test *p*-values, and effected size, determined as Cohen’s d, in sensor-derived features before/after intervention
N. Caballol et al.	^88^	Treatment monitoring	Analyzing the responsivity of sensor measure in detecting ON-OFF time, dyskinesia, Freezing	Significant differences, determined by statistical test *p*-values, in sensor-derived features between medication changed group and medication stable group. Kappa agreement analysis to evaluate the agreement between clinical interview and sensor results.
I. Thomas et al.	^89^	Intervention decision	Evaluating the accuracy of a sensor-based medication dosing schedules (SBDS)	Correlation and mean relative errors between sensor prediction and clinical experts’ evaluation
A. Silva de Lima et al.	^33^	Patient stratification	Evaluating Fall incidence rate in PD vs Controls using IMU wearable sensors	Significant differences, determined by statistical test *p*-values, in sensor-derived features across different patient populations
M. Mancini et al.	^46^	Patient stratification	Analyzing difference in turning between Freezers and not Freezer using IMU wearable sensors	Significant differences, determined by statistical test *p*-values, in sensor-derived features across different patient populations
L. Haertner et al.	^90^	Patient stratification	Evaluate difference in gait parameters in PD with and without Fear of Falling using IMUs sensors	Significant differences, determined by statistical test *p*-values, in sensor-derived features across different patient populations
K. Srulijes et al.	^34^	Patient stratification	Evaluating fall incidence rate in different disease population using IMU wearable sensors	Significant differences and correlations of sensor-derived features with patient diagnosis and clinical scores
M. Marano et al.	^43^	Patient stratification	Evaluating fall incidence rate in different patient population using IMU wearable sensors	Significant differences, determined by statistical test *p*-values, in sensor-derived features across different patient populations
M. Mancini et al.	^45^	Patient stratification	Automated detection of Freezing of Gait using IMU sensors data and detection of freezers vs non-freezers	Significant differences, determined by statistical test *p*-values, in sensor-derived features across different patient populations. Accuracy of FOG events detected compared with clinical rating
K. Kyritsis et al.	^91^	Patient stratification	Training a machine learning algorithm to distinguish PD vs Control from IMU sensor data during eating	AUC performance of machine learning algorithm in predicting patient diagnosis from sensor derived measures
A. Atrsaei et al.	^44^	Patient stratification	Evaluating the effect of fear of falling on sensor derived gait parameters	Significant differences, determined by statistical test *p*-values, in sensor-derived features across different patient populations
A. Mirelman et al.	^92^	Patient stratification, Disease prediction	To assess sleep disturbances in different PD vs Healthy Controls using objective sensor measures	Significant differences, determined by statistical test *p*-values, in sensor-derived features across different patient populations
V. Shah et al.	^32^	Disease prediction	To determine which sensor-derived mobility measures of discriminate PD from healthy control	AUC performance of machine learning algorithm
L. Adams et al.	^26^	Disease prediction	Evaluating difference in sensor-derived mobility parameters in PD vs Huntington patients	Significant differences, determined by statistical test *p*-values, in sensor-derived features across different disease populations
V. Shah Al.	^31^	Disease prediction	Evaluating difference in sensor-derived gait parameters in PD, Multiple Sclerosis and Healthy Controls.	Significant differences, determined by statistical test *p*-values, and AUC in sensor-derived features across different patient populations.
A. Nouriani. et al.	^24^	Disease prediction	Evaluate the predictive value of sensor-derived features for prospective fall frequency.	Linear regression analysis, between sensor-derived features and fall frequency prediction, rho, standard and mean squared error, t-stat and *p*-values are reported.
L. Evers et al.	^30^	Disease prediction, Symptom monitoring	To monitor motor fluctuations in PD using sensor derived measure during gait segment and to discriminate against healthy controls.	AUC performance of machine learning algorithm in predicting patient conditions and diagnosis from sensor derived measures.
H. Gaßner et al.	^41^	Symptom monitoring	To evaluate the reliability of supervised, standardized sensor-based gait outcomes at home compared to the hospital.	Significant differences, determined by statistical test *p*-values and intra class correlation coefficients, between sensor-derived features at home and in the lab.
L. Zhu et al.	^48^	Symptom monitoring	Correlating patient conditions with discrepancy between PROMs and Objective measures.	Correlation of sensor derived information with self-reported outcomes, rho and p-value. Statistical analysis with *p*-values and intra class correlation coefficients.
A. Rodríguez- Molinero et al.	^38^	Symptom monitoring	To investigate the link between gait sensor derived features and UPDRS III.	Correlation of sensor derived information with clinical scores, rho and *p*-value.
A. Rodríguez- Molinero et al.	^47^	Symptom monitoring	To investigate the link between sensor-derived gait measures ON-OFF state.	Accuracy and positive/negative predictive values of machine learning algorithm results based on sensor-derived features with patient-reported outcomes.
A. Lígia Silva de Lima et al.	^36^	Symptom monitoring	To investigate the link between motor fluctuations and sensor derived parameters.	Linear regression analysis, between sensor-derived features and clinical scores, rho and *p-*value, *R*^*2*^ before and after levodopa intake.
B. Boroojerdi et al.	^27^	Symptom monitoring	Evaluating correlation between total motor activity during sleep measured through sensors and patient reported sleep quality.	Descriptive statistic between sensor-derived features and patient reported outcomes. Difference between at home and at clinic behavior.
M. Knudson et al.	^93^	Symptom monitoring	Predicting from objective sensor measures activity of daily life impairment.	*R*^*2*^ performance of multiple regression algorithm, based on sensor-derived features, when predicting clinical scores. Significant differences in sensor values for patients with and without bradykinesia and dyskinesia, *p-*values reported.
A. Rodríguez-Molinero et al.	^67^	Symptom monitoring	Estimate dyskinesia severity from sensor-derived measurements.	Correlation of sensor derived information with Unified Dyskinesia Rating Scale, rho and *p*-value and confidence intervals.
Ravichandran. et al.	^22^	Symptom monitoring	Evaluate if sensor derived sensor derive features are sensitive to ON-OFF state.	Descriptive statistic of sensor derived value in different states.
A. Papadopoulos et al.	^94^	Symptom monitoring	Detecting tremor episode using smartphone IMU during phone calls.	Average and standard deviation of: Precision, Sensitivity, Specificity, F1-score performance of machine learning algorithm.
M. Heijmans et al.	^61^	Symptom monitoring	To evaluate symptom severity of people with PD through wearables and digital questionnaires.	AUC performance of logistic regression algorithm on a patient used as case study.
D. Gatsios et al.	^60^	Symptom monitoring	Feasibility study aiming to collect relevant clinical data in the wild.	Significant differences and correlations between sensor use and clinical scores, rho and *p*-value.
R. San-Segundo et al.	^95^	Symptom monitoring	Detecting tremor episode from sensor-derived data using deep learning algorithms.	AUC performance and False Positive Rate at 0.90 True Positive Rate of machine learning algorithm in predicting patient symptom severity
A. Abrami et al.	^21^	Symptom monitoring	To evaluate correlation between movement symbolic representation and clinical scores.	Significant differences and correlations between of sensor derived information with clinical scores, rho and *R*^*2*^.
R. Bouça-Machado et al.	^62^	Symptom monitoring	Feasibility study aiming to collect relevant clinical data in the wild via wearables.	Correlation of sensor derived information with clinical scores, rho and *p*-value.
M. Corrá et al.	^35^	Symptom monitoring	Evaluate if unsupervised sensor derived gait speed and walking bout duration detect ON-OFF state.	Significant differences and correlations between of sensor derived information with clinical scores, rho, *p*-value and *R*^*2*^.
Y. Raykov et al.	^23^	Symptom monitoring	To predict from gait sensor data if segment recorded happened before-after medication intake.	Mean and standard error in predicting patient conditions from sensor derived measures.
J. Habets et al.	^96^	Symptom monitoring	To detect significant difference in wrist IMU data during bradykinesia fluctuations and predicting them with a machine learning algorithm.	Significant differences, determined by statistical test F, Wilk’s Lambda and *p*-values. AUC performance of machine learning algorithm in predicting patient conditions from sensor derived measures
G. Oyama. et al.	^42^	Symptoms monitoring	To evaluate the test-retest reliability in the home environment of a digital sensor-based assessment. Correlation with in lab clinical scores.	Evaluation test-retest reliability across different study periods, determined by Intra class correlation coefficients. Spearman correlation between sensor measure at home and clinical scores at lab.
M. Hssayeni et al.	^68^	Symptom monitoring	To continuously predict UPDRS III from sensors recordings.	Correlation, determined by rho and *p*-value, and mean absolute errors between sensor prediction and clinical scores.
F. Lipsmeier et al.	^37^	Symptoms monitoring	Monitoring patient condition at home using a combination of unsupervised tasks and passive monitoring using objective sensor measures.	Spearman correlation, determined by rho and *p*-value, between sensor derived feature and clinical scores. Intra class correlation coefficients, evaluating test-retest reliability across different study periods. Significant differences, determined by statistical test *p*-values.
M. Burq et al.	^40^	Symptoms monitoring	Monitoring patient condition at home using a combination of unsupervised tasks and passive monitoring using objective sensor measures.	Correlation, determined by rho and confidence intervals, between sensor prediction and clinical scores. Intra class correlation, evaluating test-retest reliability across different study periods.
D. Safarpour et al.	^39^	Symptoms monitoring	Evaluating rigidity, postural instability and gait difficulties from wearables measures.	Correlation, determined via multivariable linear regression by rho and *p*-value, between sensor prediction and clinical scores measured in the lab.
FS. Kanellos. et al.	^51^	Symptom monitoring	Investigate the correlation between at lab and at home clinical assessment.	Correlation, determined by rho and *p*-value, between sensor prediction and clinical scores. Bland-Altman test between sensor prediction and clinical score.
JL. Adams. et al.	^50^	Disease prediction, Symptoms monitoring, Progression monitoring	Evaluate differences in digital features between early PD and controls. Links of sensor features and patient symptom severity.	Significant differences, determined by statistical test *p*-values, in sensor-derived features across different disease populations and patient conditions.
R. Powers et al.	^25^	Symptom monitoring, Progression monitoring, Intervention decision	Monitoring motor symptom severity using objective sensor measures.	Correlation of sensor derived information with clinical scores, rho and *p*-value. Significant differences, determined by statistical test *p*-values, in sensor-derived features across different patient conditions and populations. Accuracy comparison between sensor prediction and clinician’s expectation.

**Table 3:** Types of Evidence provided in the EEL-III

Authors study	Reference	Aim	Target	Clinical utility evidence provided
D.A. Heldman, et al.	^52^	Clinical decision support	Evaluating the impact of sensor derived motor symptom severity information on clinical decision	Qualitative description of sensor contribution in the clinical decision. Number of patients influenced. Changes in sensor-derived clinical scores before-after treatment modification
A. Hadley et al.	^53^	Clinical decision support	Evaluating the impact of sensor derived motor symptom severity information on clinical decision and treatment evaluation	Qualitative description of sensor impact in the clinical decision. Significant differences, determined by statistical test *p-*values, in sensor measures before/after treatment change.
S. Isaacson et al.	^54^	Clinical decision support	Evaluating through a randomize intervention study if sensor-based information improves motor symptom management	Least Square mean improvement in clinical score UPDRS II and III, linked to *p*-value. Mean medication change and mean number of dosage change between sensor -supported clinical decision group and standard clinical decision group.
A. Santiago et al.	^55^	Clinical decision support	Investigating the impact of continuous recording from PKG on clinical decisions.	Qualitative evaluation of sensor contribution in the clinical decision. Number of patients influenced.
P. Farzanehfar et al.	^56^	Clinical decision support	Evaluating the efficacy of detecting wearing off through sensor data and the clinical impact on patients	Significant differences, determined by statistical test *p*-values, in clinical score total UPDRS and III between sensor -supported clinical decision group and standard clinical decision group.
R. Powers et al	^25^	Clinical decision support	Analyzing the impact of sensor derived motor symptom severity information on clinical decision	Qualitative evaluation of sensor contribution in the clinical decision. Number of patients influenced. Accuracy of decision based on sensor alone vs standard procedure.
V. De Cock et al.	^57^	Personalized treatment	Evaluating the effect of an individualized gait training treatment based on sensor derived parameters	Significant differences, determined by statistical test *p*-values, in clinical scores before/after intervention.
T. Chomiak et al.	^58^	Personalized treatment	Evaluating the effect of an individualized gait training treatment based on sensor derived parameters	Significant differences, determined by statistical test *p*-values, in sensor-derived features across intervention and control group.
H. Gaßner et al	^59^	Personalized treatment	Evaluating the effect of an individualized gait training treatment based on sensor derived parameters	Significant differences, determined by statistical test *p*-values, in sensor-derived features, clinical scores and patient-defined motor tasks before/after intervention.

### User Experience

Twenty-one articles included user experience outcomes about the usability of wearables or participants’ adherence. Two articles investigated exclusively whether the patients considered a device usable^26,27^, and 11 articles focused only on adherence to the study protocol^19,25,32,37,48,54,55,57,58,60,61^, and 8 evaluated both^18,20,22,40,52,57,59,62^.

The duration of remote data collection across the different studies spanned from 2 days to 16 months. The small sample size of the cohort was the most frequent limitation, with only two papers that enrolled >50 participants.

The largest study that assessed usability and adherence was from Lima et al., which included 953 PD participants in North America (NAM) and the Netherlands (NL), monitored for 6 or 13 weeks^20,33,36^. Overall, 84% of participants contributed sensor data, although this amount was affected by the platform usability score and self-reported user depression. Participant’s adherence to the study protocol ranged from 62% (14.8 h/participant/day) up to 68% (16.3 h/participant/day) in the different countries. A general decreasing trend in time for adherence was observed in both cohorts, with the daily accelerometer data recording a reduction of 23% in the NL after 13 weeks and 27% in NAM after 6 weeks.

Burg et al. reported on 388 people with early-stage PD where usability was measured as the participant’s ability to perform the protocol test during the in-clinic visit, obtaining almost a perfect score (100% for tremor and upper extremity bradykinesia and 98.5% for gait)^40^. Adherence was evaluated in terms of median wear-time (21.1 h/day), and the percentage of per-protocol remote assessments completed was 59%. Similar to the previous study, the adherence rate decreased with time from 80% of participants who had at least one virtual motor exam during the first week to just 40% in week 52, with a dropout rate of 5.4%.

In general, all papers reviewed presented good results regarding adherence and usability. Participants reported positive experiences with wearables and found them easy to use and incorporate into their daily routines. Nevertheless, long-term data collection still measured a substantial decrease in adherence with time. Detailed user experience results are summarized in Supplementary Table 4.

## Discussion

The primary aim of this manuscript was to assess the evidence of clinical utility and general usability of wearables for home-monitoring applications to understand if these are two critical elements that limit the translation of research results into the patient’s journey in real-world healthcare scenarios. This was evaluated through a systematic review, which categorized the level of maturity of wearable technologies in research studies by assigning tailored Efficacy Evidence Levels for each paper.

Most manuscripts published in literature on wearable sensors in PD focus exclusively on technology development under a laboratory test environment. Consequently, they were excluded from this review during the screening phase. However, we could identify 59 manuscripts that transfer wearable technology into home monitoring, which adds to the technical measurement aspects, the real-world context, and non-standardized clinical assessments.

The division of the articles with our framework highlighted how the majority (>80%) is oriented toward technical efficacy or diagnostic sensitivity. Consequently, it does not directly generate evidence of improvement in health outcomes, diagnosis, treatment management, or prevention. Only nine articles could be categorized into the EEL-III, clinical utility level. Two major clinical contexts were associated with these studies. The authors either presented an improvement in patients’ condition after sensor-based training interventions, or demonstrated the utility of wearables for healthcare professionals during their clinical decisions.

The interventional studies testing the effect of wearables technologies revealed that sensors could play an essential role in this type of trial with closed-loop feedback, individualized training parameters, and primary outcome measures. The crucial factor observed was training intensity. Protocols with a minimum training frequency of 150 min per week for at least 6 weeks yielded considerably higher benefits than a lower training frequency^63^. These findings align with previously published research on rehabilitation training^64^.

When investigating clinical decision support, the primary aim of digital technologies was to provide doctors with a better overview of the individual patient’s motor symptom severity and fluctuation. This allowed a personalized treatment selection and a more accurate evaluation of the therapy effects in time. However, the final impact of sensors on health outcomes has been assessed quantitatively only in two studies. In the work from A. Farzanehfar et al.^56^, sensor-based therapy modification led to a decrease of 6 MDS-UPDRS III and 12 total MDS-UPDRS points compared to regular assessment. Similarly, Isaacson et al.^54^ observed a significant least square mean improvement of 2.6 points in UPDRS II and a descriptive improvement of 4 points in UPDRS III. The remaining four studies in the EEL-III group presented qualitative results on clinical utility.

This imbalance between quantitative vs. qualitative results highlights a critical gap in the assessment of clinical utility. In the future, it would be essential having harmonized conditions and study designs to assess and compare the clinical utility of the different digital technologies. Ideally, for the specific case of Parkinson’s, the wearables effect should be evaluated on long-term disease progression, rapid changes due to interventional trials, and standard-of-care treatment cycles^65^.

A better evaluation framework for clinical utility of studies would lead not only to an improved care for patients, but also to validated metrics to assess wearables outcomes and support clinical decision-making. It is important mentioning in this regard, the results of a recent scoping review on the clinical adoption of gait analysis technologies (which was not specific to the clinical and usability settings covered in this manuscript)^66^. This work identified as major barriers to adoption of sensors, as reported by healthcare professionals, the underlying interpretability of the measurements in the clinical decision context, and the lack of literacy available with reference data to establish objective comparisons. These findings highlight the necessity of more numerous and structured clinical utility studies.

Within the diagnostic sensitivity category EEL-II, the studies reviewed presented a broad range of targeted motor symptoms within heterogeneous applications, participants, and sample sizes.

Studies converge in claiming the feasibility of *“symptoms monitoring”* and *“treatment monitoring”*: continuously tracking in the home environment clinically relevant information about the severity of patient motor symptoms^25,30,35,37,47,61,62,67^. This tracking is affected by “Real-World” noise, i.e., movements from unsupervised daily activities mimicking PD symptoms, which may lead to errors in the sensor report and false alarms. A robust strategy to filter this type of interference still needs to be found.

Also, studies consistently agree that measurements in the unsupervised environment are not just an extension of what is observed in the lab but a new perspective that substantially differs. Nonetheless, links and correlations were found across the two settings^21,25,41,68^, highlighting the importance of having lab measurements and unsupervised scripted tasks in the home environment as a reference.

When investigating patients’ stratification, wearable sensors demonstrated sufficient sensitivity to capture variations between inter and intra-disease^69^. Combined with artificial intelligence, sensor-derived data and digital outcomes can discriminate PD vs. HC and different PD patient sub-types, such as fallers vs. non-fallers^43,44^ or freezers vs. non-freezers^45,46^. Long-term, digital outcomes could be essential in diagnosing and identifying potentially harmful patient risk factors. However, especially in this field, it is essential to mention how the proper evaluation of the results should revolve primarily on clinical utility efficacy aspects rather than the pure numerical performance of the algorithm. It must be proven that these smart models can still provide additional value to healthcare professionals in real-world applications, which present additional constraints compared to a research environment.

Surprisingly, only a few articles included analyses associated with non-motor symptoms despite the complex multidimensional nature of Parkinson’s disease. A possible explanation is the additional hardware and software required to evaluate the non-motor sphere (e.g., symptom questionnaires, patient diaries, or qualitative interviews), consequently limiting the number of validated devices on the market that monitor motor and non-motor symptoms. This gap represents a huge growth opportunity for the future, particularly if we consider the extreme relevance of non-motor symptoms from a clinical perspective and their significant impact on the quality of life of people with PD.

The articles in the *Technical efficacy* group focused on proving feasibility or identifying movements and segments of interest, such as walking periods, whose characteristics can be linked to clinically relevant information in follow up studies. This category was the smallest as expected. In fact, more controlled and less noisy environments are usually preferred when evaluating this type of research questions. All the works in this category showed promising results. In the work of Reykov et al.^23^, walking segments in the unsupervised environment were detected with a specificity and sensitivity of 91% for PD patients. Excellent performance was achieved also in the study of Nouriani et al.^24^ where walking, standing, and turning have been detected with an accuracy of above 99%. However, a common limitation was the small sample size, with the biggest study that enrolled 25 people with PD.

In parallel to evidence, in this manuscript, we evaluated *“User experience”* through *adherence* and *usability*. Overall, wearables received a high usability rating and were well accepted by the patients in the different cohorts. However, the duration of the studies evaluated was limited in some cases^26^^,^^27^, which calls for long-term evaluation of these aspects.

Furthermore, the drop in the adherence in time remains a challenge to be addressed in future work. In the context of wearable technologies, the potential bias due to the ‘novelty effect’ must be considered^70^. However, reasons for discontinuation of use need to be systematically identified and tackled, including, e.g., privacy concerns, lack of digital health literacy^71^, motivations behind unwillingness of long-term/continuous use^72,73^. Family problems can also play a role. In this case, also caregivers need to be included in the overall usage concept of technical devices, allowing for flexibility given heterogenous personal care needs. To achieve long-term adherence and fit to people’s everyday life, patient-centered approaches to design and evaluate body worn devices are highly recommended, e.g., through co-design and value-based studies^74^, taking into consideration needs for informed decision-making and autonomy^75,76^.

Finally, it is important to highlight that only 21 out of 59 articles provided usability or adherence outcomes, most of which were feasibility studies with small sample sizes. Furthermore, measurement methods for user experience were highly variable across articles, with a limited number using standardized usability evaluations and the rest using self-developed, non-validated user questionnaires. For this reason, it is essential to consistently introduce usability assessments as a regular practice, irrespective of the cohort under investigation or the type of study undertaken, while using systematic criteria for evaluating and iterating on user experience and by conducting both quantitative and qualitative research in a systematic way^17^. Patient engagement strategies and a patient-centered design for user experience should be explicitly implemented for long data acquisition periods to mitigate the drop in concordance/adherence observed in many longitudinal projects. In future work, an iterative approach to designing and evaluating a holistic user experience likely prevent expensive changes to mature prototypes that showed low adherence in clinical studies.

To complete this discussion section, it is important to highlight also two main limitations of the current work, which are essential to the correct interpretation of the clinical utility results of this manuscript. First, part of the devices eventually integrated within standard of care procedures, are developed completely inside the manufacturer validation pipeline. Consequently, their development does not generate research studies that can be evaluated in this review.

**Fig. 4:**
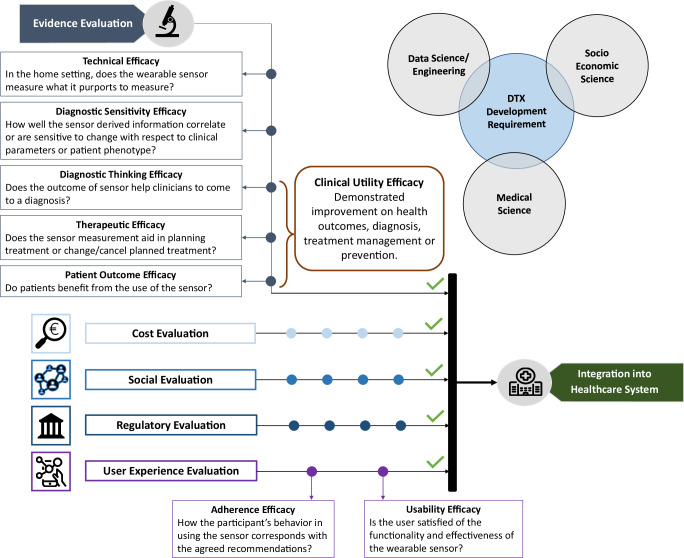
Multi-perspective evidence model. Multi-perspective model to evaluate the maturity level of wearable technologies toward a standard of care integration, with different sciences contributing to translating research concepts into clinical care.

Second, our EEL framework was primarily developed to categorize the paper screened in this review. A comprehensive and accurate evaluation of the clinical utility of wearables would require a *“Health Technology Assessment”* approach that explores multiple domains in parallel to evidence and user experience, see Fig. 4. Additionally, this evaluation should be carried not in research studies, as performed in this manuscript, but within the multiple Real-Word clinical pathways of the people with PD. However, this was beyond the scope of our manuscript.

Recently, the (state-of-the-art) management strategy of PD started a profound transformation following the advent of Precision Medicine. Generic treatments have been replaced by individualized interventions tailored on the necessities of the single patient, and subjective evaluations substituted by objective data driven assessments. The combination of innovative technologies and algorithms, such as wearable sensors and machine learning models, triggered this change making new types of information available to healthcare professionals. This has impacted the decision-making process, which has shifted toward a “Quadruple Decision-Making Model” that is not anymore exclusively based on professional expertize, but combines expert opinion, patient preferences, scientific evidence, and big data approaches^76^.

At the same time, this transformation is slowly but steadily shifting the role itself of wearables technologies. They are not expected anymore to simply replicate of what is done in regular hospital assessments. They are requested to provide a different perspective and added value. In summary, they need to provide clinical utility evidence.

The results of this manuscript, shows that wearable sensors, such as PKG, MM4PD, Kinesia 360, Ambulosono among others (see also the work of C). Moreau et al. for an anthology of the most used body-warn sensors for PD^13^, can already play a relevant role as clinical decision support tools for neurologists or during individualized rehabilitation programs. Consequently, the low amount of clinical utility evidence observed in the research papers screened, seems to be motivated more by a lack of study designs investigating these factors, rather than the technological readiness of the systems. To maximize the potential of wearable sensors and their positive impact on the care strategy of PD is essential investigate deeper the clinical utility of these technologies and to generate validated reference metrics that can be used in the different care pathways.

Since the introduction of new devices within the medical procedures often comes with changes in professional roles and tasks^77^, new types of specialized trainings will be required (as was the case in articles reviewed^50^), and as offered by professional healthcare networks in some countries^78^. Firstly, for healthcare professionals to use novel devices and integrate the sensor information into their care routines. Secondarily, for patients to learn how to properly handle these new tools.

To conclude, this manuscript presents a review of the clinical utility evidences of wearable sensors in research studies focusing on people with PD in the home environment. The results showed that within the large production of articles centered on this topic, only a very limited number of studies generate evidence about clinical utility. This phenomenon could explain, in part, the gap between technical readiness and usage within standard of care that is observed for wearable technologies in PD.

As already mentioned, analyzing the limitations of the EEL framework, the fully comprehensive evaluation of the impact of wearables in PD management would require a more complex and multi perspective approach. However, it is important to consider that research trials, thanks to the possibility of shaping the study designs and selecting inclusion/exclusion criteria of the participants, offer a more controlled and suitable environment to investigate pure clinical utility. They can create the ideal conditions to limit the interference of external factors while maximizing the impact of wearable tools on healthcare outcomes. For this reason, they play a vital role in the generation of clinical utility evidence.

Ultimately, the results of our work strongly suggest that the scarcity of clinical utility evidence is induced primarily by the lack of study designs tailored to quantify improvement in health outcomes, and not by existing technical hardware/software limitations. Wearables nowadays can generate accurate and relevant diagnostic information (as shown by the EEL-II) that, if used, clearly provides a benefit to patients and doctors (EEL-III). For this reason, we encourage future researchers to focus more on clinical utility studies, generating results that will shape the future care strategies for Parkinson’s Disease.

## Methods

**Fig. 5:**
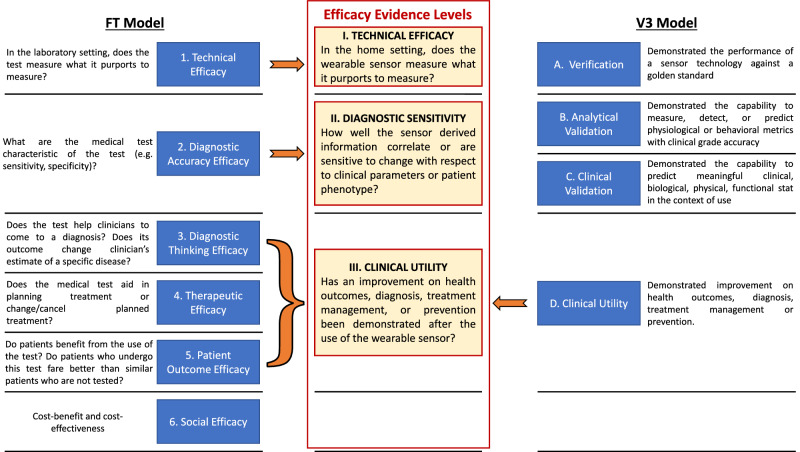
Evidence level frameworks. Framework for Efficacy Evidence Levels (EELs) utilized for categorizing the articles within this manuscript. The EEL model is presented together with the Fryback and Thornbury^15^ and the V3 models^16^ in order to facilitate the comparison across different frameworks.

To evaluate and assign the selected research articles to different maturity levels based on the given evidence, we generated an *“Efficacy Evidence Level”* model (EEL) tailored towards the three essential milestones wearables need to achieve. They have to undergo a prove of technical precision and accuracy, a validation of their in home-based applications, and the proof of clinical utility for their measures or parameters, i.e., being beneficiary for individual patients and/or their healthcare professionals. The EEL model builds on two evaluation frameworks with a more general comprehensive evaluation scope (Fig. 5). In 1991, Fryback and Thornbury (FT) proposed a model to categorize diagnostic tests in general, where their maturity was classified into six levels of efficacy, spanning from *technical efficacy* if the test correctly measures what it is supposed to in a laboratory setting, up to *social efficacy*, where cost-benefit and cost-effectiveness have to be proven^15^. Adapted versions of this model have been utilized in more recent works, such as the 2020 review by K. van Leeuwen et al., who classified AI-based commercially available products in radiology^79^. In 2020, a different approach was proposed by Goldsack et al.^16^: the V3 framework evaluates the maturity level of biometric monitoring technologies according to 4 validation classes and presents *Clinical Utility* as the maximum level of maturity, i.e., if it has been demonstrated an improvement on health outcomes, diagnosis, treatment management or prevention.

For feasibility reasons, we tailored the evaluation items of the two models to the clinical utility of wearables for home monitoring in PD. Thus, our framework is composed of three hierarchical EELs defined as follows:

1. *Technical efficacy*: the first EEL evaluates the accuracy of the information being measured by the sensor.
2. *Diagnostic sensitivity:* the second EEL analyzes how well sensor-derived information correlate or are sensitive to change compared to clinical parameters or patient phenotype. Four application domains can be foreseen: distinguishing across different conditions, stratifying across patients intra-disease, evaluating symptoms severity, and evaluating changes in patient conditions on long or short timeframes (disease progression and treatment cycles, respectively).
3. *Clinical utility:* the third level of EEL considers to which extent the data measured by the sensors can be translated into practical knowledge supporting personalized clinical decisions and positive effects on the individual patient’s clinical outcome.

The definitions for the first two levels were inspired by the FT model, whereas the clinical utility EEL was adjusted to the V3 clinic grade accuracy scale, as the latter fits better to evaluate the evidence of wearable technologies from feasibility studies.

Together with the evidence level, a separate analysis focusing on user experience has been performed. User Experience can be independently evaluated at different levels of maturity. In fact, multiple reviewed articles from all the EELs investigated these aspects. For this reason, user experience has been analyzed in parallel to the primary evidence efficacy framework.

### Article selection and structured reviewing procedure

The systematic review of the articles was performed according to the PRISMA principles^17^. The research was conducted using the PubMed Central database (RRID: SCR_004846) and included studies with publication dates between January 2017 and May 2023. Only original, full-text articles published in English that described the usage of wearable sensors for home monitoring of people with PD were included in the review. All types of body worn technologies were considered for this review. However, manuscripts based exclusively on surveys without a real usage of devices were excluded. Specific search terms were used for a detailed literature review: (Parkinson*) AND (measure OR monitoring) AND (free-living OR daily living OR continuous OR 24-h OR home OR unsupervised) AND (sensors), located within the title, abstract, or full text.

The article filtering process was composed of two phases. First, text availability, titles, and abstracts were checked as a preliminary screening of the extracted links. Second, the full texts of the selected manuscripts were further analyzed to include articles for final review.

The modified AXIS appraisal tool method was utilized to analyze the risk of bias^80,81^. Each article was scored between 0 and 13, summing the number of positive answers in the reviewer’s assessment (see Supplementary Table 5). Papers with scores below 10 points were considered at medium-high risk of bias and consequently excluded from the review.

Five independent reviewers (GZ, MJ, OT, AC, SS) selected and evaluated the studies. In case of disagreement, the articles were discussed with non-reviewing co-authors. To be included in the review, the article had to meet the criteria listed below:

### Inclusion criteria

- Type of paper: original, full-text, peer-reviewed, journal or articles
- Time frame: January 2017–May 2023
- Participants: people with PD
- Environment for assessment: Home monitoring
- Information source: wearable sensors

### Exclusion criteria

- Type of paper: conference articles.
- Studies conducted in artificial “home-like” environments.
- Reviews, meta-analyses, and papers not reporting original data.
- Article only describes the protocol or study design without concrete results.
- Article without access to the full-text version.
- Articles with an AXIS score below 10 points.
- Surveys without usage of wearables
- No body-worn sensors

### EEL-framework categorization

Finally, the articles included were categorized according to the EEL framework based on their research questions. Papers investigating pure technical precision, without correlating sensor-derived measures with any medical parameters, were assigned to the *“Technical Efficacy”* group, EEL-I. In the EEL-II, *“Diagnostic Sensitivity”*, were included studies that applied statistical analysis, algorithms, or machine learning models to link sensor outcomes with clinical scores and patient profiles, cross-sectionally or longitudinally. Finally, in the *“Clinical Utility”* grade, EEL-III, were assigned papers where sensor-derived measures were utilized to enhance patient care. This was achieved either by improving decision-making from healthcare professionals through relevant information or actively tailoring interventions according to the device measurements.

Studies were assigned to more than one category when they presented multiple research questions associated with the different levels of our framework. Detailed results from the reviewed articles are shown in separate tables related to each level of the EEL framework (Supplementary Tables 1, 2 and 3).

### User experience evaluation

In addition to categorizing the 3 EELs, we evaluated the user experience aspects presented in the reviewed studies. *User Experience* has been considered by reviewing *adherence* and *usability* results^62,69^. The WHO defines *adherence* as “the degree to which the person’s behavior corresponds with the agreed recommendations from a healthcare provider” and measured regarding data contribution and task completion rates^82^. According to ISO norms, *usability* is defined as “the degree to which a given user’s goal is achieved in terms of effectiveness, efficiency, and satisfaction”^83^. These two aspects are positively correlated and vital when considering the implementation and diffusion of remote monitoring wearable sensors in healthcare.

## Supplementary information

Supplemental Material

## Data Availability

The data source utilized to generate Fig. 1 and the PRISMA diagram are available in Zenodo repository (REF: 10.5281/zenodo.11349002).
