# Supplementary material for: Assessing the clinical utility of inertial sensors for home monitoring in Parkinson’s disease: a comprehensive review
Source: NPJ Parkinsons Dis. 2024 Aug 20;10:161. doi: 10.1038/s41531-024-00755-6 (PMC11335938; doi:10.1038/s41531-024-00755-6)
Supplement: Supplementary file 1 — Supplemental Material [file 41531_2024_755_MOESM1_ESM.pdf]

Supplementary Materials

Supplementary Table 1. Technical Efficacy level results

Abbreviations: PD = Parkinson's Disease HC = Controls or Healthy Controls, OA= Older Adults, NPH = Normal Pressure Hydrocephalus, MDS-UPDRS- Movement Disorder Society-Unified Parkinson's Disease Rating Scale, AUC: Area Under the Curve, Controlled environment: Laboratory/Clinic/Hospital under the supervision of a clinician/researcher.

Authors Study	Year	Reference	Type	Aim	Outcome Measure	Study Population	Symptoms / Domains	Position	Analysis	Results	
**A. Abrami et al.**	2020	[21]	6 IMU (APDM Wearable Technologies)	Feasibility testing	Movement decomposition in 'syllables'	35 PD @ Lab - 25 PD @ home - 45 HC	Bradykinesia, activities of daily life, mobility	Upper and Lower Extremities, Trunk	Clustering of the movements in different syllables composition. Analyzing the differences between HC and PD - movement decomposition and clinical scores.	Controlled environment	Applying a linear regression model to the multi-dimensional scaling algorithm values. MDS-UPDRS-III score estimation with high accuracy ($p < 0.05$, $r^2 = 0.54$)
										Home environment	Average MDS-UPDRS-III score correlates well with diurnal and nocturnal activities.
**Y. Raykov et al.**	2021	[23]	6 IMUs	Algorithm Validation	Gait recognition, Medication-induced gait fluctuation	25 PD - 25 HC	Mobility	Upper and Lower extremities, Trunk	Detection of walking segments in an unsupervised environment. Binary classification of the gait segment before and after medication.	Controlled environment	-
										Home environment	The walking segments were detected with both specificity and sensitivity above 90%. The maximum balanced accuracy obtained in the binary classification was 76%.
**M. Ullrich et al.**	2021	[28]	2 IMUs (Mobile GaitLab)	Algorithm Validation	Gait recognition, Gait Speed	12 PD	Mobility	Lower extremity	Automated detection and decomposition of unsupervised standardized gait tests during continuous home recording.	Controlled environment	-
										Home environment	The unsupervised standardized gait test was detected with an F1-score of 88.9% and decomposed with an F1-score of 94%.
**Y. Brand et al.**	2022	[29]	4 IMUs (Protokinetics)	Algorithm Validation	Gait recognition	18 PD - 12 non-PD	Mobility	Upper extremities	Automated detection of gait segments through different algorithms.	Controlled environment	-
										Home environment	The anomaly detection algorithm achieved an AUC of 0.80 and 0.74 for Non-PD and PD. The best-performing detection algorithm, a deep convolutional neural network, exhibited high AUCs (i.e., 0.94 for OAs and 0.89 for PD).
**Ravichandran et al.**	2023	[22]	2 IMU (Flexpoint)	Feasibility Testing	Fine finger movement	4 PD	Tremor, bradykinesia, dyskinesia	Upper extremities	Frequency and Peak Analysis of raw signals. Followed by feature extraction through Random Forest classifier.	Controlled environment	-
										Home environment	Correlation of sensor-derived features against MDS-UPDRS III motor scores. Significance of movement features were tested against various classifiers with Random Forests performing best with a precision, recall, and accuracy of > 0.70.
**A. Nouriani et al.**	2023	[24]	5 IMU (Soarkfun, InvenSense)	Algorithm Validation	Physical activity recognition	11 PD - 8 NPH - 10 HC	Activities of daily life, Risk of falls	Lower extremities, Trunk	Estimation of limb segment orientation through a non-linear sensor fusion algorithm	Controlled environment	-
										Home environment	Walking, standing, and turning has been detected with the highest accuracy (>99%). Low likelihood events have been detected with high sensitivity >95% with the exception of near-falls events which was 80%.

Supplementary Table 2. Diagnostic Sensitivity level results

Abbreviations: PD = Parkinson's Disease, HC = Controls or Healthy Controls, MS = Multiple Sclerosis, HD = Huntington's Disease, PSP = Progressive Supranuclear Palsy, MDS-UPDRS: Movement Disorder Society-Unified Parkinson's Disease Rating Scale, TUG: Time-up and Go, H&Y: Hoehn & Yahr, FoG: Freezing of Gait, ROC: Receiver Operating Curve, AUC: Area Under the Curve, ICC: Intraclass Correlation Coefficient, PIGD: Postural Instability and Gait difficulties. LR- levodopa response, FOF – fear of falls, UDysRS- Unified Dyskinesia Rating Scale, NPH-Normal Pressure Hydrocephalus, Controlled environment: Laboratory/Clinic/Hospital under the supervision of a clinician/researcher.

Authors Study	Year	Reference	Type	Aim	Outcome Measure	Study Population	Symptoms Monitored	Position	Analysis	Results	
**R. Bhidayasiri et al.**	2017	[84]	1 IMU (NIGHT-recorder)	Treatment monitoring	Number of turns in bed and degree of axial turn	34 PD	Sleep	Trunk	Statistical analysis between intervention and control group.	Controlled environment	Significantly greater improvements in clinical scale-based assessments in intervention group - from baseline to end of treatment in patients given rotigotine vs. placebo.
										Home environment	Significant difference in the number of turns in bed (ANCOVA, p=0.001), and degree of axial turn (p=0.042) between rotigotine vs placebo.
**A. Rodríguez-Molinero et al.**	2017	[38]	1 IMU	Symptom monitoring	Frequency content of strides	75 PD	Mobility	Trunk	Correlation analysis between sensor-derived features and MDS-UPDRS scores.	Controlled environment	-
										Home environment	Moderate correlation of sensor-derived features with the MDS-UPDRS-III score (rho −0.56; p < 0.001). Correlation between the algorithm outputs and the gait item (3.10) of the MDS-UPDRS-III was good (rho −0.73; p < 0.001).
**M. Iijima et al.**	2017	[85]	1 IMU (PGR)	Treatment monitoring	Walking profiles	14 PD	Mobility	Trunk	Statistical analysis of sensor-derived features before and after the addition or increase in dose of Selegiline	Controlled environment	-
										Home environment	The MDS-UPDRS III and FoG scores significantly improved after the addition or increase in dose of selegiline (p < 0.005, p < 0.01, respectively). However, changes in gait-related scores of MDS-UPDRS were not detected in six patients.
**L. Adams et al.**	2017	[26]	5 IMUs (BioStampRC)	Disease prediction	Activities of daily life, mobility	16 PD - 15 HD - 5 prodromal HD - 20 HC	Activities of daily life	Upper and Lower extremities, Trunk	Statistical analysis of daily living activity across different types of groups.	Controlled environment	-
										Home environment	Individuals with HD spent more than 50% of the time lying down, substantially more than individuals with prodromal HD (33%, p = 0.003), PD (38%, p = 0.01), and HC (34%; p < 0.001).
**M. Mancini et al.**	2018	[46]	1 IMU (McRoberts, DynaPort Hybrid)	Patient stratification	Quality metrics of turning	94 PD	Mobility	Trunk	Statistical analysis of sensor-derived features during turning in freezers vs non-freezers.	Controlled environment	-
										Home environment	The number of turns (19.3±9.2/30 min in freezers, 22.4±12.9/30 min non-freezers) was similar in the two groups. Mean jerkiness, mean and variability of mediolateral jerkiness were significantly higher in the freezers, compared to non-freezers. Subjects with FoG showed differences in the turns with larger angles compared to those without FoG.
	2018	[47]	1 IMU	Symptom monitoring		23 PD	Mobility, bradykinesia,	Trunk		Controlled environment	-

**A. Rodríguez-Molinero et al.**					Gait, bradykinesia		motor fluctuations		Comparison between sensor-detected and self-reported bradykinesia.	Home environment	The algorithm produced 37% more results than the patients' records (671 vs 489). The positive predictive value of the algorithm to detect OFF-periods, as compared with the patients' records, was 92% and the negative predictive value was 94%; the overall classification accuracy was 92.20%.
**A. Lígia Silva de Lima et al.**	2018	[36]	1 IMU	Symptom monitoring	Mean time spent walking	304 PD	Mobility	Upper extremities	Correlation analysis between motor fluctuation and average daily walking quantity before and after levodopa intake.	Controlled environment	-
										Home environment	Participants spent most of their time walking between 8 am and 1 pm. The severity of motor fluctuations did not influence the mean time spent walking, but older age and greater severity of motor symptoms was associated with less time spent walking.
**L. Haertner et al.**	2018	[90]	1 IMUs (RehaGait)	Patient stratification	Turning parameters	55 PD	Mobility	Lower back	Statistical analysis of turning parameters accordingly to 4 different patient characterizations.	Controlled environment	Fear of Falling was associated with a longer turning duration and lower maximum and middle angular velocities of turns.
										Home environment	Lack of fear of falling was associated with lower maximum and average angular velocities of turns. The positive history of falls was not associated with turning parameters, neither in the lab nor in the home.
**H. Khodakarami et al.**	2019	[86]	1 IMU (PKG)	Treatment monitoring	Response to levodopa	199 PD - 191 HC	Bradykinesia, dyskinesia, tremor	Upper extremities	Machine learning model to estimate if sensor-derived features can predict LR measured by MDS-UPDRS	Controlled environment	-
										Home environment	The ROC AUC of the model trained on all the subjects was 0.80. Substantial improvement (ROC AUC = 0.92) was obtained by excluding those participants in the uncertain MDS-UPDRS range.
**K. Srulijes et al.**	2019	[34]	4 IMUs (activPAL™)	Patient stratification	Physical activity, Incidence of falls	14 PD - 31 DA - 12 PSP - 31 HC	Mobility	Lower extremities	Statistical analysis between physical activity and incidence of falls	Controlled environment	-
										Home environment	Absolute fall incidence was high in all three patient groups, with differing levels (DA - 9 falls; PD - 14 falls; PSP - 29 falls per year). Correction for individual exposure to physical activity revealed that low walking activity was associated with higher incidence of falls.
**B. Boroojerdi et al.**	2019	[27]	4 IMUs (NIMBLE patches)	Symptom monitoring	Accelerations, muscle activity, usability of the system	27 PD	Bradykinesia	Upper and Lower extremities, Trunk	Correlation between sensor predicted scores and rater evaluation of MDS-UPDRS.	Controlled environment	The overall correlation coefficient between rater evaluation and sensor algorithm-predicted scores of MDS-UPDRS was 0.471 (p = 0.031). Rest tremor amplitude and toe-tapping had the highest correlations (0.746 and 0.709 respectively).
			with Surface EMG							Home environment	No discernable relationship was identified between sensor-derived features (the total amounts of motor activity, total time lying down during sleep) and patient-reported (via Diary app) quality of the sleep
**I. Thomas et al.**	2019	[89]	1 IMU (PKG)	Intervention decision	Sensor-based medication dosing schedules (SBDS)	25 PD	Bradykinesia, dyskinesia, motor fluctuations	Upper extremities	Correlation analysis between clinician and sensor-predicted medication dosing schedules .	Controlled environment	-
										Home environment	The sensor-based dosing system (SBDS) maintenance and morning dosing suggestions had a Pearson's correlation of 0.80 and 0.95 (with mean relative errors of 21% and 12.5%), to the sensor-guided dosing

											adjustments. Paired t-test indicated no statistical differences between the algorithmic suggestions and the clinician's adjustments.
**A. Rodríguez-Molinero et al.**	2019	[67]	1 IMU	Symptom monitoring	Sensor-derived dyskinesia severity	13 PD	Dyskinesia, motor fluctuations	Trunk	Correlation analysis between sensor-derived features and the Unified Dyskinesia Rating Scale (UDysRS), administered by a physician.	Controlled environment	-
										Home environment	The correlation coefficient between the sensor output and UDysRS score was 0.70 (CI 95%: 0.33–0.88; p = 0.01). The correlation between the sensor output and UDysRS sub-items of (the trunk and legs) was 0.91 (CI 95% 0.76–0.97: p < 0.001).
**A. Papadopoulos et al.**	2019	[94]	1 IMU	Symptom monitoring	Tremor detection during phone calls	31 PD<br>-<br>14 HC	Tremor	Upper extremities	Identification of tremor episodes using machine learning.	Controlled environment	-
										Home environment	The models showed high AUC performance on the different testing datasets.
**M. Heijmans et al.**	2019	[61]	1 IMU (MOX5)	Symptom monitoring	Tremor, dyskinesia, usability of the system	20 PD	Bradykinesia, dyskinesia, tremor, motor fluctuations	Upper extremities	Statistical analysis on user adherence. Machine learning model to predict ON/OFF state based on sensor-derived features	Controlled environment	-
										Home environment	Participants used wearable sensors 94% of the instructed timeframe. High questionnaire completion rates (79,1%). A logistic regression classifier was able to detect off-state moments with an AUC = 0.73.
**V. Shah et al.**	2020	[32]	3 IMU (Opals APDM)	Disease prediction	43 digital outcome measures of mobility	29 PD<br>-<br>27 HC	Activities of daily life, mobility	Upper and Lower extremities, Trunk	Logistic regression to predict PD vs HC	Controlled environment	-
										Home environment	Six digital outcome measures of mobility identified with AUC>0.80. Turn angle (AUC=0.89, 95% CI: 0.79–0.97) and swing time variability (AUC=0.87, 95% CI: 0.75–0.96) were the most discriminative features.
**M. Knudson et al.**	2020	[93]	1 IMU (PKG)	Symptom monitoring	Bradykinesia and dyskinesia severity, usability of the system	34 PD	Bradykinesia, dyskinesia, Activities of daily life	Upper extremities	Statistical analysis on self-reported and sensor-derived symptom severity. Regression model to predict MDS-UPDRS II score from sensor-derived features and answer to the questionnaire.	Controlled environment	-
										Home environment	The highest reduction of bradykinesia occurred 30 min after the medication intake. Average change of bradykinesia severity (sensor-derived measures), overall dyskinesia, and patients-reported symptoms score were used to create a significant multiple regression model to predict UPDRS part II score.
**A. Mirelman et al.**	2020	[92]	1 IMU (Axivity AX3)	Patient stratification, Disease prediction	Lying, turning, and upright time at night	305 PD<br>-<br>205 HC	Bradykinesia	Trunk	Statistical analysis on sensor-based features and disease duration.	Controlled environment	-
										Home environment	Nocturnal lying time was similar among all groups (HC, 7.5 ± 1.2 hours; H&Y stage 1, 7.3 ± 0.9 hours; H&Y stage 2, 7.2 ± 1.3 hours; H&Y stage 3, 7.4 ± 1.6 hours; P = 0.501). Patients with advanced PD had more upright periods, the number and velocity of their turns were reduced (P ≤ 0.021).
**A. Silva de Lima et al.**	2020	[33]	1 IMU		Number of falls	2063 PD<br>-	Mobility	Neck		Controlled environment	-

			with barometer	Patient stratification		2063 HC			Statistical analysis on Incidence ratio of Falls in PD vs HC	Home environment	The incidence rate of any fall was higher among PD patients than HC (2.1 vs. 0.7 falls/person). The incidence rate of a new fall after enrollment (i.e., hazard ratio) was 1.8 times higher for PD patients than HC.
**V. Shah Al.**	2020	[31]	3 IMUs (Opals APDM)	Disease prediction	Gait parameters	16 PD - 15 MS - 31 HC	Mobility	Lower extremities, Trunk	Statistical analysis of sensor-derived features across 3 groups	Controlled environment	The toe-off angle best discriminated MS versus HC (AUC= 0.95). The Lumbar coronal range of motion best discriminated PD versus HC (AUC=0.78).
										Home environment	The gait speed best discriminated MS versus HC in daily life (AUC= 0.84). The foot-strike angle best discriminated PD versus HC (AUC= 0.84).
**H. Gaßner et al.**	2020	[41]	2 IMUs (Shimmer)	Symptom monitoring	Gait parameters, UPDRS III scores	20 PD	Mobility	Lower extremities	Difference in MDS-UPDRS and scripted gait task in Lab vs at Home	Controlled environment	-
										Home environment	MDS-UPDRS-III and TUG were comparable at Lab versus at Home. PD patients' gait at home was slower, strides were shorter, and shuffling of gait was more present. Narrow walkways (<85 cm) resulted in a significant reduction of gait velocity at home. Reliability analysis at Lab vs at Home revealed excellent ICC coefficients for MDS-UPDRS-III and sensor-derived gait parameters.
**L. Evers et al.**	2020	[30]	8 IMUs (Gait Up SA, Motorola Moto 360 Sport, Empatica E4)	Disease prediction, Symptom monitoring	Power spectral density of accelerometer signals	18 PD - 24 HC	Mobility	Upper and Lower extremities, Trunk	Binary classification of gait segments before and after the medication. Classification of gait segment in PD vs HC.	Controlled environment	-
										Home environment	The binary classifier achieved a maximum performance of 0.84 AUC in predicting outcome before vs after medication. A 0.76 AUC was reached in differentiating between PD vs Controls.
**L. Zhu et al.**	2020	[48]	1 IMU (WIMU-GPS)	Symptom monitoring	Real Life community mobility	54 PD	Mobility	Trunk	Comparing objective sensor-derived features vs self-reported mobility.	Controlled environment	-
			1 IMU with GPS							Home environment	Convergent validity was attained by the sensor for trip frequency (rs = .69, 95% confidence interval [CI] = 0.52–0.81) and duration outside (rs = .43, 95% CI = 0.18–0.62), but not for life space size (rs = .39, 95% CI = 0.14–0.60).
**D. Gatsios et al.**	2020	[60]	4 IMUs (Moticon, Aquaris U and M, Microsoft Band)	Symptom monitoring	Accelerations, pressure distribution, cardiac activity, usability of the system	75 PD	Tremor	Upper and Lower extremities	Regression analysis on sensor-derived features and clinical scores	Controlled environment	-
			4 IMUs with 2 pressure sensors							Home environment	Patients used the system for a median 11.57 (SD 3.15) days. Statistically significant, strong positive correlation between the tremor severity and amplitude and constancy of tremor as evaluated at baseline by the clinicians and a moderate positive correlation with tremor as perceived by the patient.
**R. San-Segundo et al.**	2020	[95]	2 IMUs (Axivity AX3)	Symptom monitoring	Upper limb Accelerations, UPDRS-III	12 PD @ Lab - 6 PD @ Home	Tremor	Upper extremities	Tremor prediction using different machine learning models.	Controlled environment	Convoluted neural network- T/NT + multi-layer perceptron leads to the highest performance in PD tremor detection.
										Home environment	CNN-T/NT + MLP leads to the highest performance in PD tremor detection @Home. The classifier trained on @Lab data performed better than the one trained with @Home data.

**A. Abrami et al.**	2020	[21]	6 IMUs (APDM wearable Technologies)	Symptom monitoring	Movement decomposition in 'syllabes'	35 PD @Lab - 25 PD @Home - 45 HC	Bradykinesia, Activities of daily life, mobility	Upper and Lower extremities, Trunk	Clustering of the movements in different syllables composition. Analysis of differences between controls and PD. Correlation of movement decomposition and clinical scores	Controlled environment	After applying a linear regression model to the multi-dimensional scaling algorithm values, it was possible to estimate the MDS-UPDRS-III score with high accuracy ($p < 0.05$, r-squared = 0.64).
										Home environment	Diurnal and nocturnal activities were well correlated with the average MDS-UPDRS-III score.
**R. Bouça-Machado et al.**	2021	[62]	1 IMU (mkinetikos)	Symptom monitoring	Compliance and system usability	20 PD	Activities of daily life, mobility	Phone-embedded	Statistical analysis of system usability and compliance to the study protocol	Controlled environment	-
			1 IMU with GPS							Home environment	Seventeen participants (85%) completed the study. Sixteen participants (94.1%) showed medium-to-high compliance with the system. A 6-point drop in the total score of the Post-Study System Usability Questionnaire was observed. Six sensor metrics correlated with clinical outcomes from moderate to strong correlation (r= 0,51-0,69, p<0,001).
**M. Corrá et al.**	2021	[35]	4 IMU (RehaGait, Physilog® 5)	Symptom monitoring	Gait speed	39 PD	Mobility, motor fluctuations	Lower extremities	Correlation of Gait Speed in ON and OFF state @Lab and @ Home. Correlation of sensor-derived features with MDS-UPDRS III score	Controlled environment	-
										Home environment	Gait speeds differentiated ON and OFF states in the lab and the home. ON assessments in the lab showed moderate-to-high correlations with faster gait speeds in unsupervised environment ($r = 0.69$; $p < 0.001$), associated with long walking bouts. OFF gait assessments in the lab showed moderate correlation values with slow gait speeds during OFF state at home ($r = 0.56$; $p = 0.004$), associated with short walking bouts.
**M. Marano et al.**	2021	[43]	1 IMU (Encephalog app)	Patient stratification	''Time Up and Go'' derived gait parameters	33 PD	Mobility	Phone-embedded	Statistical analysis of smartphone-derived gait parameters in fallers vs non-faller group	Controlled environment	-
										Home environment	The stand-up time and the mediolateral sway differed between "fallers" and "non-fallers". In a multivariate model, the stand-up time and the history of falling independently related to the outcome.
**Y. Raykov et al.**	2021	[23]	6 IMUs (HopkinsPD app, Physilog 4)	Symptom monitoring	Gait recognition, medication - induced fluctuations	25 PD - 25 HC	Mobility	Upper and Lower extremities, Trunk	Detection of walking segments in an unsupervised environment. Binary classification of the gait segment before and after medication.	Controlled environment	-
										Home environment	The walking segments were detected with both specificity and sensitivity above 90%. The maximum balanced accuracy obtained in the binary classification was 76%.
**M. Mancini et al.**	2021	[45]	5 IMUs (Opals APDM)	Patient stratification	Freezing episodes, time spent freezing, freezing variability	40 PD at Lab - 48 PD at Home	Mobility	Lower extremities, Trunk	Prediction of Freezing episodes based on sensor signals	Controlled environment	Moderate to good correlation between the number of FoG episodes detected in the laboratory by clinician and sensor-based evaluation for short and long FoG.
										Home environment	The total time of freezing episodes and the variability of total time differentiated between people with and without FoG ($p < 0.05$) Short FoG episodes account for 69% of all FoG episodes.
**R. Bouça-Machado et al.**	2021	[87]	1 IMU (Axivity AX3)	Treatment monitoring	Gait Parameters, Clinical scores	24 PD	Mobility	Trunk	Statistical analysis of sensor-derived features before and after the specialized multidisciplinary intervention.	Controlled environment	-
										Home environment	Step length (from supervised TUG with Cognitive test), and free-living step time asymmetry were identified as the best predictors of TUG changes and detected a small to moderate effect of the intervention.

**K. Kyritsis et al.**	2021	[91]	1 IMU	Patient stratification	Plate-to-Mouth indicator	7 HC + 21 PD @Lab - 7 HC + 13 PD @Home	Non-motor symptoms	Upper extremities	Machine learning model to differentiate PD vs HC	Controlled environment	The model achieved an AUC of 0.748 for differentiating PD vs HC in lab.
										Home environment	The model achieved an AUC of 0.775 for differentiating PD vs HC in home environment.
**A. Atrsaei et al.**	2021	[44]	3 IMUs (Mobility Lab APDM)	Patient stratification	Falls, gait parameters	26 PD	Mobility	Lower extremities, Trunk	Statistical analysis of gait parameters in people with fear of falling (FOF) and without FOF	Controlled environment	The peak angular velocity of the turn-to-sit transition of the TUG test had the highest discriminative power between people with FOF and without FOF.
										Home environment	People with FOF demonstrated lower gait speed at home and a lower amount of walking bouts for shorter walking bouts.
**J. Habets et al.**	2021	[96]	1 IMU (Gait Up Physilog 4)	Symptom monitoring	Kinematic metrics	20 PD	Bradykinesia	Upper extremities	Statistical analysis on kinematic metrics. Machine learning prediction of bradykinesia fluctuations.	Controlled environment	-
										Home environment	Statistically significant AUCs for medication-induced bradykinesia fluctuation classification were seen in 85% of PD patients.
**R. Powers et al.**	2021	[25]	1 IMU (MM4PD)	Symptom monitoring, Progression monitoring, Intervention decision	Tremor and dyskinesia severity	343 PD cross-sectional - 225 PD longitudinal	Dyskinesia, motor fluctuations, tremor	Upper extremities	Correlation of sensor-derived features with clinical scores. Usability of sensor reports for clinical-decision support.	Controlled environment	Sensor-derived features correlated with clinical evaluations for tremor severity (ρ = 0.80) and mapped to expert ratings of dyskinesia presence (P < 0.001).
										Home environment	Sensor-derived motor fluctuations and tremor severity showed a good correlation with patients' self-reported symptoms and prescribed medication time. Symptom changes captured sensor matched clinician's expectations in 94% of cases. In the remaining 6% of cases, the data identified opportunities to make clinically applicable treatment changes.
**M. Hssayeni et al.**	2021	[68]	2 IMUs	Symptom monitoring	Gyroscope-derived features, MDS-UPDRS III	24 PD	Motor fluctuations	Upper and Lower extremities	Machine learning model to predict MDS-UPDRS-III scores based on sensor-derived features.	Controlled environment	-
										Home environment	The estimated MDS-UPDRS-III scores had a correlation of 0.79 (p < 0.0001) and a mean absolute error of 5.95 with the clinical examination scores.
**F. Lipsmeier et al.**	2022	[37]	2 IMUs (Moto G 360 2nd Gen Spor)	Symptoms monitoring	Bradykinesia, speech, tremor, mobility, cognitive function	316 PD	Bradykinesia, mobility, tremor, non-motor symptoms	Upper extremities	Evaluation of system adherence and test–retest reliability. Correlation analysis between sensor-derived features associated to motor, speech, cognitive levels based on MDS-UPDRS scores.	Controlled environment	-
										Home environment	Average system adherence was high (96.29%). All pre-specified sensor features demonstrated good-to-excellent test–retest reliability between the first two 2-week study periods ([ICCs] ≥ 0.75). Correlations with MDS-UPDRS item scores revealed that all sensor features correlated with their corresponding clinical items.
**M. Burq et al.**	2022	[40]	1 IMU (Verily)	Symptoms monitoring	Bradykinesia tremor, mobility	388 PD	Bradykinesia tremor, mobility	Upper extremities	Correlation analysis between sensor-derived features and MDS-UPDRS scores. System Adherence.	Controlled environment	Analytical validation was established for in-clinic measurements, which showed moderate-to-strong correlations with consensus of MDS-UPDRS Part III ratings for rest tremor (ρ=0.70), bradykinesia (ρ=−0.62), and gait (ρ=−0.46).
										Home environment	Median wear-time was 21.1 h/day, and 59% of per-protocol remote assessments were completed. Remote measurements were sensitive to the known effects of dopaminergic medication (ON vs OFF Cohen's d=0.19–0.54).
**D. Safarpour et al.**	2022	[39]	IMU (Opals APDM)	Symptoms monitoring	Gait and balance parameters	31 PD	Mobility	Lower extremities, Trunk	Correlation analysis between clinical scores and sensor-derived gait parameters	Controlled environment	-
										Home environment	The number of walking bouts and turns correlated significantly with the rigidity sub-score, while the number of turns, foot pitch angle, and sway area while

											standing correlated significantly with the PIGD sub-score. The rigidity sub-score was best predicted by the number of walking bouts while the PIGD sub-score was best predicted by a combination of number of walking bouts, gait speed, and postural sway.
**JL. Adams et al.**	2023	[50]	6 IMUs (Opals APDM, Apple watch, BrainBaseline app)	Disease prediction, Symptoms monitoring, Progression monitoring	Gait parameters	50 HC - 82 PD	Mobility, Fine Motor Function, Tremor, Speech, Cognition	Upper and Lower extremities, Trunk	Correlations across sensor-derived features and clinical evaluation	Controlled environment	-
										Home environment	Strong correlation across gait parameters derived from smartwatch and smartphone (0.36 < r < 0.79) vs. research-grade IMUs. Significant differences in finger tapping were found in dominant vs. non-dominant side in PD vs HC. Tremor fraction strongly correlated to MDS-UPDRS III sub-item 17 (Rest Tremor Amplitude) r = 0.86, p < 0.001 and sub-item 18 (Constancy of Rest Tremor) r = 0.79, p < 0.001.
**FS. Kanellos et al.**	2023	[51]	5 IMUs (PDMonitor)	Symptom Monitoring	UPDRS-III estimation, Quality of Life	20 PD	Bradykinesia, Tremor, FoG, Dyskinesia	Upper extremities, Trunk	Validity of lab-based MDS-UPDRS rating vs. home monitoring (Pearson's correlations)	Controlled environment	Sensor-derived bradykinesia, rigidity, gait and balance impairment, moderately correlated to clinical MDS-UPDRS ratings.
										Home environment	Sensor-derived bradykinesia (r = 0.62), dyskinesia, and tremor (r = 0.79) moderately correlated in the home environment to MDS-UPDRS rating. Indicative of daily symptom fluctuation not observed by clinical scores. The time in "OFF" period correlates with MDS-UPDRS II score (r = 0.49) and moderately with PDQ-8 score (r = 0.73).
**G. Oyama et al.**	2023	[42]	1 IMU	Symptom monitoring	Virtual Motor Examination	100 PD	Motor Function	Upper extremities	Validity of sensor measurements at home vs lab.	Controlled environment	Clinical assessment was superior to single-sensor data collection for bradykinesia (|r|= 0.63, reliability = 0.72), tremor (|r|= 0.41, reliability = 0.65), and overall motor features (|r|= 0.7, reliability = 0.67)
										Home environment	Virtual evaluation reliability ranged between 0.62 and 0.8 for weekly averages, with significantly lower reliability for daily scores 0.24–0.66.
**N. Caballol et al.**	2023	[88]	1 IMU (STAT-ON)	Treatment monitoring	FoG, Changes to MDS-UPDRS	39 PD	Percentages of OFF-Time, ON-Time, dyskinesia, and FoG	Trunk	Univariate comparisons across groups with and without treatment adjustment. Agreement analysis for symptoms. Spearman correlation for MDS-UPDRS score.	Controlled environment	-
										Home environment	An improvement on MDS-UPDRS III score with significant differences across OFF/ON times in treatment group. Agreement across number of steps and FoG ((p = 0.008 and 0.004 respectively) after levodopa modifications. Sensors detected more ON-Time in the patients in whom levodopa was increased (p = 0.003). Number of FoG events increased in group without the treatment, no changes in treatment group (p = 0.004)
**Ravichandran et al.**	2023	[22]	2 IMUs (Flexpoint)	Symptom monitoring	Fine finger movement	4 PD	Tremor, bradykinesia, dyskinesia	Upper extremities	Frequency and Peak Analysis of raw sensor signals. Feature extraction through Random Forest classifier.	Controlled environment	-
										Home environment	Correlation of sensor-derived features with MDS-UPDRS III scores. Significance of movement features were tested against various classifiers with Random Forests performing best with a precision, recall, and accuracy of > 0.70.
**A. Nouriani et al.**	2023	[24]	5 IMUs (Soarkfun, InvenSense)	Disease prediction	Activity recognition	11 PD - 8 NPH - 10 HC	Activities of daily living, Risk of falls	Lower extremities, Trunk	Estimation of limb segment orientation through a non-linear sensor fusion algorithm.	Controlled environment	-
										Home environment	Walking, standing, and turning has been detected with the highest accuracy (>99%). Low likelihood events had sensitivities of >95%, for near-falls events - 80%.

Supplementary Table 3. Clinical Utility level results

Abbreviations: PD = Parkinson's Disease, HC = Controls or Healthy Controls, FES-I= Fall Efficacy Scale-International, MoCA- Montreal Cognitive assessment test, FOG-Q – Freezing of Gait Questionnaire, PDQ-39 – Parkinson's Disease Questionnaire – 39, MDS-UPDRS - Movement Disorder Society- Unified Parkinson's Disease Rating Scale, Controlled environment: Laboratory/Clinic/Hospital under the supervision of a clinician/researcher.

Authors Study	Year	Reference	Type	Aim	Outcome Measure	Study Population	Symptoms Monitored	Position	Analysis	Results	
**D.A. Heldman, et al.**	2017	[52]	1 IMU (Kinesia)	Clinical decision support	Tremor, bradykinesia, dyskinesia, system compliance	18 PD	Bradykinesia, dyskinesia, tremor	Upper extremities	Statistical analysis on motor symptom progression between intervention vs control group.	Controlled environment	-
										Home environment	Finger-tapping speed improved with increasing levodopa dose (r = -0.68, p = 0.04). There was no significant correlation between changes in dyskinesia severity and levodopa dose. Effective therapy adjustment using system report for 2 participants.
**T. Chomiak et al.**	2017	[58]	1 IMU	Personalized treatment	Step height	11 PD	Mobility	Lower extremities	Statistical analysis on the clinical scores and motor automaticity between intervention and control group.	Controlled environment	-
			microphone for audio stimuli							Home environment	No significant effect of training was observed on FES-I, MoCA, or FOG-Q. A significant group difference (music vs podcast) was observed by training interaction in dual-task step automaticity (P<0.01).
**S. Isaacson et al.**	2019	[54]	1 IMU (Kinesia 360)	Clinical decision support	Sensor-derived scores of motor symptom severity	40 PD	Bradykinesia	Upper and Lower extremities	Statistical analysis of longitudinal changes in MDS-UPDRS between experimental and control group.	Controlled environment	-
										Home environment	At week 12, least squares mean improvements in MDS-UPDRS II (-2.1 vs 0.5, p = 0.004) and MDS-UPDRS III (-5.3 vs -1.0, p = 0.134) were clinically meaningful. Mean rotigotine dosage higher (4.8 vs 3.9 mg/24 h) in experimental vs control group.
**A. Santiago et al.**	2019	[55]	1 IMU (KinetiGraph)	Clinical decision support	Utility of system-derived features	89 PD	Bradykinesia, dyskinesia, motor fluctuations	Upper extremities	Survey-based analysis to evaluate the utility of sensor measures for adjusting the treatment.	Controlled environment	-
										Home environment	In 143 clinical studies with 89 PD patients, 46 out of 112 physician surveys (41%) found the sensors provided useful additional information for adjusting the treatment. In 66 surveys (59%), the sensors didn't provide new information.
**V. De Cock et al.**	2021	[57]	1 IMU (BeatWalk)	Personalized treatment	Walking test parameters, usability of the system	45 PD	Mobility	Lower extremities	Statistical analysis of walking test parameters after 4 weeks of gait training.	Controlled environment	During the 6 minutes walking test an improvement was observed in distance, cadence, velocity, stride length.
										Home environment	Patients used the application 78.8% of the prescribed duration. The patients showed a lower number of falls during the gait training program than they typically experienced in a week (0.22 ± 0.76 vs. 0.11 ± 0.46, p= 0.07).
**A. Hadley et al.**	2021	[53]	1 IMU (KinesiaU)	Clinical decision support	Tremor, bradykinesia, dyskinesia, usability of the system	16 PD	Bradykinesia, dyskinesia, tremor	Upper extremities	Clinical recommendation based on sensor reports.	Controlled environment	-
										Home environment	The sensor-based assessments successfully captured intra-day fluctuations and short/long-term responses to therapies, including detecting significant improvements (p < 0.05) in at least one symptom in 7 participants.
**P. Farzanehfar et al.**	2021	[56]	1 IMU (KinetiGraph)	Clinical decision support	Bradykinesia	200 PD	Motor fluctuations	Upper extremities	Statistical analysis on wearing-off in	Controlled environment	-
										Home environment	Sensor measurement can successfully detect the wearing-off periods. Participants with wearing-off

									clinical ratings vs sensor rating.		periods experienced worse motor and non-motor symptoms and lower quality of life. Quality of life significantly improved when wearing-off was treated.
**R. Powers et al**	2021	[25]	1 IMU (MM4PD)	Clinical decision support	Tremor and dyskinesia severity	343 PD cross-sectional - 225 PD longitudinal	Motor fluctuations, dyskinesia, tremor	Upper extremities	Correlation of sensor measurements with clinical features. Usability of sensor reports for clinical decision support.	Controlled environment	Sensor measurements correlated with clinical evaluations of tremor severity (ρ = 0.80) and dyskinesia (P < 0.001).
										Home environment	Motor fluctuations and tremor severity showed a good correlation with patients' self-reported symptoms. Symptom changes captured sensor matched clinician's expectations in 94% of cases. In the remaining 6% of cases, the data identified opportunities to make clinically applicable changes in medication strategy.
**H. Gaßner et al.**	2022	[59]	1 IMU (SHIMMER 2)	Personalized treatment	Individualized training	15 PD	Bradykinesia	Lower extremities	Statistical analysis of longitudinal changes in UPDRS, Timed Up and Go Test; 2-minute walk test and PDQ39	Controlled environment	-
										Home environment	87% of patients (13/15) reported 40% (p<0,001) improvement in daily motor tasks. No significant impact was noted on the quality of life as assessed by the Parkinson's Disease Questionnaire (but the subsections regarding mobility and social support improved by 14% and 19% respectively). Motor symptoms rated by MDS-UPDRS part III, did not improve significantly but a descriptive improvement of 14% from 18 to 16 could be observed.

Supplementary Table 4. User Experience level results

Abbreviations: PD = Parkinson's Disease, HC = Controls or Healthy Controls, HD = Huntington's Disease, NL- Netherlands, NAM- North America.

Authors Study	Year	Reference	Type	Aim	Outcome Measure	Study Population	Data Collection	Position	Analysis	Results	
**T. Chomiak et al.**	2017	[58]	1 IMU	Personalized treatment	Step height	11 PD	4-week prospective intervention study	Lower extremities	Analysis on adherence	Usability	-
			microphone for audio stimuli							Adherence	The average total usage – 20 minutes per week (74±52minutes). Patients were trained for an average of 5.4±2.9 days, and 8.4±4.3 minutes per session. The music group trained slightly more (89 minutes, 5.8 days, 7.7 min/session) than the podcast group (61 minutes, 5.2 days 9.1 min/session), there was no statistically significant difference (t 0.89; P≥0.39 for all tracking measures).
**A. de Lima et al.**	2017	[20]	1 IMU (Pebble smartwatch)	Adherence and compliance	Sensor derived movement features. Medications	953 PD	from 6 weeks up to 13 weeks	Upper extremities	Enrolment rate, compliance	Usability	Data contribution was not affected by demographics, clinical characteristics, or attitude towards technology, but was by the platform usability score in the Netherlands (NL) ($\chi2$ (2) = 32.014, p<0.001), and self-reported depression in North America (NAM) ($\chi2$(2) = 6.397, p = .04).
										Adherence	Enrolment rate was 88% in the NL (n = 304) and 51% (n = 649) in NAM. Overall, 84% (n = 805) of participants contributed sensor data. Participants were compliant for 68% (16.3 hours/participant/day) of the study period in NL and for 62% (14.8 hours/participant/day) in NAM. Daily accelerometer data collection decreased 23% in the NL after 13 weeks, and 27% in NAM after 6 weeks.
**L. Adams et al.**	2017	[26]	5 IMUs (BioStampRC)	Disease prediction	Activities of daily life, mobility	16 PD, - 15 HD, - 5 prodromal HD, - 20 controls	2-day of recording	Upper and Lower extremities, Trunk	Statistical analysis on acceptance	Usability	The majority of participants found the sensors to be "comfortable" (n = 42) and "easy to remove" (n = 50). The most reported complaint (n = 31) was linked to the adhesion of the sensors - sensors falling off or coming loose. 86% (n=48) of participants reported ''willing'' or ''very willing'' to use the sensors in the future
										Adherence	-
**Heldman, D. A. et al.**	2017	[52]	1 IMU (Kinesia)	Clinical decision support	Tremor, bradykinesia, dyskinesia, compliance	18 PD	1 day per week for 7 months	Upper extremities	Analysis on compliance and usability	Usability	17 out of the 18 participants completed the usability questionnaire. 70.6% were very willing to use the system in the future.
										Adherence	Median compliance in using the system at home was 95.7% with an interquartile range of 77.2–99.2%.
**S. Cohen et al.**	2018	[19]	2 IMU (Apple and Pebble smartwatches)	Adherence and acceptance	Compliance levels	51 PD - 17 HD	6 months monitoring period	Upper extremities	Analysis on compliance levels and their progression	Usability	-
										Adherence	None (− 1%) to a 30% reduction in compliance rate was registered for HD patients, and a reduction of 34% to 53% was registered for the PD patients. Both groups showed marked changes in compliance rates during the initial days of enrollment. Daily sensor data streaming patterns were similar in both groups - peaking around noon, dropping sharply in the late evening hours around 8 pm, and having a mean of 8.6 h daily streaming for the PD group and 10.5 h for HD group.

**B. Boroojerdi et al.**	2019	[27]	4 IMUs (NIMBLE patches)	Symptom monitoring	Movement patterns, muscle activity, usability of the system	6 PD for Study I - 21 PD Study II	Total 3 days (2 in the clinic, 1 at home)	Upper and Lower extremities, Trunk	Participants' feedback on usability	Usability	Participants found the sensors easy to use, not painful to detach, not interfering with daily activities or sleep, nor causing embarrassment in public and generally very satisfied with the training provided. Most of the participants reported the information collected with the sensor to be helpful and considered the diary app as an easy-to-use tool.
			with Surface EMG							Adherence	-
**M. Heijmans et al**	2019	[61]	1 IMU (MOX5)	Symptom monitoring	Tremor, dyskinesia, usability of the system	20 PD	2 weeks of monitoring	Upper extremities	Statistical analysis on adherence and questionnaire completion rate	Usability	-
										Adherence	Participants wore sensors for 94% of the timeframe. Questionnaire completion rates were high (79.1%)
**S. Isaacson et al.**	2019	[54]	1 IMU (Kinesia 360)	Clinical decision support	System derived scores of motor symptom severity	40 PD	12 weeks in total. (Min 2 consecutive days in Weeks 1, 2, 3, 4, and 11)	Upper and Lower extremities	Analysis on wearing times	Usability	-
										Adherence	Participants in the experimental group used the device for a mean of 29.2 days (median: 26.0 days). Participants who used the device for a longer duration showed a slightly greater increase in rotigotine dosage during the study than participants who used the device for shorter duration
**A. Santiago et al.**	2019	[55]	1 IMU (KinetiGraph)	Clinical decision support	Physicians' survey	89 PD	6 or more days of recording.	Upper extremities	Analysis on survey completion rate	Usability	-
										Adherence	Survey completion rate was 83% (n= 119 out of 143). Survey completion rates were the highest for patients with only one sensor (97%) and decreased with two sensors to 64% and to 50% with three sensors.
**A. Botros et al.**	2019	[18]	3 IMU (Axivity AX3, DomoCare)	Usability	System usability	4 PD	4 Weeks	Upper and Lower extremities, Trunk	Statistical analysis on system usability	Usability	Overall System Usability Score - 71.5%. Acceptance score of 87% for the body sensors and 100% for ambient sensors.
										Adherence	-
**V. Shah et al.**	2020	[31]	3 IMU (Opals APDM)	Disease prediction	43 digital outcome measures of mobility	29 PD - 27 HC	1 week of continuous monitoring (min 8 hours/day)	Upper and Lower extremities, Trunk	Statistical analysis on wearing time	Usability	-
										Adherence	Duration of recordings was 66±14 hours in the PD and 59±16 hours in HC
**L. Zhu et al.**	2020	[48]	1 IMU with GPS (WIMU GPS)	Symptom monitoring	Real Life community mobility	54 PD	14 days monitoring	Trunk	Analysis on diary completeness	Usability	-
										Adherence	7.7% (75/980 days) had missing or incorrect information. Significant variability of diary completion.
**D. Gatsios et al.**	2020	[60]	4 IMUs with 2 pressure sensors (Moticon, Aquaris U and M, Microsoft Band)	Symptom monitoring	Accelerations, pressure distribution, cardiac activity, usability of the system	75 PD	2 weeks of monitoring (min 12 hours per day)	Upper and Lower extremities	Analysis on drop out and wearing times	Usability	-
										Adherence	65 of 75 (87%) participants completed the study. The device was used for a median of 11.57 (SD 3.15) days. 65 participants collected data for a median of 63.37 days. 2 participants used the system for 1 day, the remaining participants used it for more than 6 days. 30 participants used the device for the entire study period – 14 days.
**R. Powers et al.**	2021	[25]	1 IMU (MM4PD)	Symptom monitoring, Progression monitoring,	Tremor and dyskinesia severity	343 PD cross-sectional -	1 week of home recording	Upper extremities	Analysis on wearing times	Usability	-
										Adherence	In the longitudinal study participants wore the device for 10.9±2.5 hours a day. Average total participation 104±59 days (μ±s). 3% drop out rate.

				Intervention decision		225 PD longitudinal					
**R. Bouça-Machado et al.**	2021	[62]	1 IMU with GPS (mKinetikos)	Symptom monitoring	Compliance and system usability	20 PD	7 months	Phone-embedded	Statistical analysis on system usability and compliance	Usability	A 6-point drop in the total score of the Post-Study System Usability Questionnaire was observed.
										Adherence	Seventeen participants (85%) completed the study. Sixteen participants (94.1%) showed a medium-to-high level of compliance with the device.
**A. Hadley et al.**	2021	[53]	1 IMU (KinesiaU)	Clinical decision support	Tremor, bradykinesia, dyskinesia, system usability	16 PD	5 weeks	Upper extremities	Analysis on drop out and wearing times	Usability	-
										Adherence	13 participants successfully completed the study and averaged 4.9 assessments per day for 3 days per week during the study Despite being requested to use the device for a minimum of 3 days in the desired weeks (9 total days), the median usage time was 12.5 days. 11 participants continued to use the system beyond the minimum use and 1 participant used the system for 30 days.
**V. De Cock et al.**	2021	[57]	1 IMU (BeatWalk)	Personalized treatment	Walking test parameters, system usability	45 PD	1-month intervention (30 minutes per day, 5 days per week)	Lower extremities	Analysis on compliance and usability	Usability	The device was considered “easy to use” by 75% of the patients.
										Adherence	Participants used the application 78.8% (±28.2) of the prescribed duration. Only 7.7% of the patients (n= 3) used the device <25% of the prescribed duration and 48.7% (n= 19) used it more than 90%. Participants performed 15.9 ± 5.8 sessions (range: 1–20) on average, out of the 20 sessions prescribed.
**F. Lipsmeier et al.**	2022	[37]	2 IMUs (Moto G 360 2nd Gen Spor)	Symptoms monitoring	Bradykinesia, speech, tremor, mobility, cognitive function	316 PD	4 weeks. Automated motor and cognitive tests	Upper extremities	Statistical analysis on adherence	Usability	-
										Adherence	On average, daily remote active testing took a median of 5.3 (interquartile range [IQR] = 1.7) minutes on days without the cognitive tests, and 7.32 (median; IQR = 1.58) minutes on days with cognitive tests. Average adherence was high with 96.29% (median per participant) of all possible active tests performed during the first 4 weeks of the study (i.e., 26–27/27 days). Participants contributed a median of 8.6 h/day of study smartphone and a median of 12.79 h/day of study smartwatch passive monitoring data.
**M. Burq et al.**	2022	[40]	1 IMU (Verily)	Symptoms monitoring	Rest tremor, bradykinesia, and Gait	388 PD	16 months follow-up. Weekly test in ON and OFF state	Upper extremities	Statistical analysis on adherence and usability	Usability	Participants’ ability to perform the test was assessed during the in-clinic visit. Participants were able to complete the required tasks -100% for tremor and upper-extremity bradykinesia and 98.5% for gait.
										Adherence	The median wear-time was 21.1 h/day. 59% of per-protocol remote assessments were completed. During the 70-week follow-up period participants completed 59% of per-protocol test sessions. The adherence rate decreased with time from 80% participants who had at least 1 virtual motor exam in the first week to 40% in week 52. The dropout rate was 5.4%.
**H. Gaßner et al.**	2022	[59]	1 IMU (SHIMMER 2)	Personalized treatment	Individualized training exercise	15 PD	4 weeks intervention.	Lower extremities	Analysis of System Usability scores and patient dropout rate	Usability	The System Usability Scale reached an average score of 72.2 (SD 6.5; 95% CI 68.5-75.8) indicating good usability of the device.
										Adherence	All participants completed the intervention phase without any dropouts.
**Ravichandran et al.**	2023	[22]	2 IMU (Flexpoint)	Feasibility Testing,	Fine finger movement	4 PD	Home monitoring for	Upper extremities	Descriptive analysis of	Usability	The device has been reported as “easy” or “very easy” to use. Time spent per session had a range

				Symptom monitoring			1 week with measured sessions after medication intake.		adherence and usability		between 5 and 15 minutes. Glove fit and wearing results were mixed across the four patients.
										Adherence	The participants used the device typically 2 times per day as instructed.

Supplementary Table 5. Modified AXIS appraisal table from Sica et Al. [82]

To analyze the risk of bias. Each article is evaluated on the 13 questions above summing the number of positive answers. Papers with scores below 10 points were considered at medium-high risk of bias and consequently excluded from the review.

Question numbers		AXIS question code
	**INTRODUCTION**	
Q1	Were the aims/objectives of the study clear?	1
	**METHODS**	
Q2	Was the study design appropriate for the stated aim(s)?	2
Q3	Was the sample size justified, clearly defined, and taken from an appropriate population?	3, 4 & 5
Q4	Was the selection process likely to select subjects/participants that were representative of the target/reference population under investigation?	6
Q5	Were the outcome variables measured appropriate to the aims of the study?	8
Q6	Were the outcome variables measured correctly using instruments/ measurements that had been trialed, piloted, or published previously?	9
Q7	Is it clear what was used to determined statistical significance and/or precision estimates? (e.g., p-values, confidence intervals)	10
Q8	Were the methods (including statistical methods) sufficiently described to enable them to be repeated?	11
	**RESULTS**	
Q9	Were the basic data adequately described?	12
Q10	Were the results presented for all the analyses described and presented in the methods?	16
	**DISCUSSION**	
Q11	Were the authors' discussions and conclusions justified by the results?	17
Q12	Were the limitations of the study discussed?	18
	**OTHER**	
Q13	Were there any funding sources or conflicts of interest that may affect the authors' interpretation of the results?	19

Preferred Reporting Items for Systematic reviews and Meta-Analyses extension for Scoping Reviews (PRISMA-ScR) Checklist

SECTION	ITEM	PRISMA-ScR CHECKLIST ITEM	REPORTED ON PAGE #
**TITLE**			
Title	1	Identify the report as a scoping review.	1
**ABSTRACT**			
Structured summary	2	Provide a structured summary that includes (as applicable): background, objectives, eligibility criteria, sources of evidence, charting methods, results, and conclusions that relate to the review questions and objectives.	2
**INTRODUCTION**			
Rationale	3	Describe the rationale for the review in the context of what is already known. Explain why the review questions/objectives lend themselves to a scoping review approach.	3-6
Objectives	4	Provide an explicit statement of the questions and objectives being addressed with reference to their key elements (e.g., population or participants, concepts, and context) or other relevant key elements used to conceptualize the review questions and/or objectives.	6
**METHODS**			
Protocol and registration	5	Indicate whether a review protocol exists; state if and where it can be accessed (e.g., a Web address); and if available, provide registration information, including the registration number.	Not registered
Eligibility criteria	6	Specify characteristics of the sources of evidence used as eligibility criteria (e.g., years considered, language, and publication status), and provide a rationale.	21 - 22
Information sources	7	Describe all information sources in the search (e.g., databases with dates of coverage and contact with authors to identify additional sources), as well as the date the most recent search was executed.	21 - 22
Search	8	Present the full electronic search strategy for at least 1 database, including any limits used, such that it could be repeated.	21 - 22
Selection of sources of evidence	9	State the process for selecting sources of evidence (i.e., screening and eligibility) included in the scoping review.	19 - 23
Data charting process	10	Describe the methods of charting data from the included sources of evidence (e.g., calibrated forms or forms that have been tested by the team before their use, and whether data charting was done independently or in duplicate) and any processes for obtaining and confirming data from investigators.	19 - 23
Data items	11	List and define all variables for which data were sought and any assumptions and simplifications made.	22 - 23
Critical appraisal of individual sources of evidence	12	If done, provide a rationale for conducting a critical appraisal of included sources of evidence; describe the methods used and how this information was used in any data synthesis (if appropriate).	21

SECTION	ITEM	PRISMA-ScR CHECKLIST ITEM	REPORTED ON PAGE #
Synthesis of results	13	Describe the methods of handling and summarizing the data that were charted.	22 – 23 and Figure 5
**RESULTS**			
Selection of sources of evidence	14	Give numbers of sources of evidence screened, assessed for eligibility, and included in the review, with reasons for exclusions at each stage, ideally using a flow diagram.	7
Characteristics of sources of evidence	15	For each source of evidence, present characteristics for which data were charted and provide the citations.	8 - 11
Critical appraisal within sources of evidence	16	If done, present data on critical appraisal of included sources of evidence (see item 12).	Figure 2 and Zenodo repository
Results of individual sources of evidence	17	For each included source of evidence, present the relevant data that were charted that relate to the review questions and objectives.	8 – 11 and Supplementary tables
Synthesis of results	18	Summarize and/or present the charting results as they relate to the review questions and objectives.	Supplementary tables
**DISCUSSION**			
Summary of evidence	19	Summarize the main results (including an overview of concepts, themes, and types of evidence available), link to the review questions and objectives, and consider the relevance to key groups.	12 - 16
Limitations	20	Discuss the limitations of the scoping review process.	17
Conclusions	21	Provide a general interpretation of the results with respect to the review questions and objectives, as well as potential implications and/or next steps.	17 - 19
**FUNDING**			
Funding	22	Describe sources of funding for the included sources of evidence, as well as sources of funding for the scoping review. Describe the role of the funders of the scoping review.	24
